# Activation of proline biosynthesis is critical to maintain glutamate homeostasis during acute methamphetamine exposure

**DOI:** 10.1038/s41598-020-80917-7

**Published:** 2021-01-14

**Authors:** Bobby Jones, Muthukumar Balasubramaniam, Joseph J. Lebowitz, Anne Taylor, Fernando Villalta, Habibeh Khoshbouei, Carrie Grueter, Brad Grueter, Chandravanu Dash, Jui Pandhare

**Affiliations:** 1 Center for AIDS Health Disparities Research, Meharry Medical College, Nashville, TN 37208 USA; 2 Center for Molecular and Behavioral Neuroscience, Meharry Medical College, Nashville, TN 37208 USA; 3 School of Graduate Studies and Research, Meharry Medical College, Nashville, TN 37208 USA; 4 Department of Microbiology, Immunology, and Physiology, Meharry Medical College, Nashville, TN 37208 USA; 5 Department of Biochemistry, Cancer Biology, Pharmacology and Neuroscience, Meharry Medical College, Nashville, TN 37208 USA; 6 Department of Neuroscience, University of Florida, Gainesville, FL 32611 USA; 7 Department of Anesthesiology, Vanderbilt University Medical Center, Nashville, TN 37232 USA; 8 Center for AIDS Health Disparities Research, Meharry Medical College, Old Hospital Bldg-CAHDR, Room 5027, 1005 Dr. DB Todd Jr Blvd., Nashville, TN 37208 USA; 9 Center for AIDS Health Disparities Research, Meharry Medical College, Old Hospital Bldg-CAHDR, Room 5023, 1005 Dr. DB Todd Jr Blvd., Nashville, TN 37208 USA

**Keywords:** Oxidoreductases, Molecular neuroscience

## Abstract

Methamphetamine (METH) is a highly addictive psychostimulant that causes long-lasting effects in the brain and increases the risk of developing neurodegenerative diseases. The cellular and molecular effects of METH in the brain are functionally linked to alterations in glutamate levels. Despite the well-documented effects of METH on glutamate neurotransmission, the underlying mechanism by which METH alters glutamate levels is not clearly understood. In this study, we report an essential role of proline biosynthesis in maintaining METH-induced glutamate homeostasis. We observed that acute METH exposure resulted in the induction of proline biosynthetic enzymes in both undifferentiated and differentiated neuronal cells. Proline level was also increased in these cells after METH exposure. Surprisingly, METH treatment did not increase glutamate levels nor caused neuronal excitotoxicity. However, METH exposure resulted in a significant upregulation of pyrroline-5-carboxylate synthase (P5CS), the key enzyme that catalyzes synthesis of proline from glutamate. Interestingly, depletion of P5CS by CRISPR/Cas9 resulted in a significant increase in glutamate levels upon METH exposure. METH exposure also increased glutamate levels in P5CS-deficient proline-auxotropic cells. Conversely, restoration of P5CS expression in P5CS-deficient cells abrogated the effect of METH on glutamate levels. Consistent with these findings, P5CS expression was significantly enhanced in the cortical brain region of mice administered with METH and in the slices of cortical brain tissues treated with METH. Collectively, these results uncover a key role of P5CS for the molecular effects of METH and highlight that excess glutamate can be sequestered for proline biosynthesis as a protective mechanism to maintain glutamate homeostasis during drug exposure.

## Introduction

Methamphetamine (METH) is a powerful and highly addictive psychostimulant^1^. It belongs to a larger group of drugs called Amphetamine-Type Stimulants (ATS)^1–3^. Use of ATS generate a sense of euphoria, increase energy and concentration as well as decrease appetite, induce weight loss, leading to a variety of emotional, cognitive, and physical effects^4,5^. Among the ATS, METH has the greatest potential for abuse because of easy availability, low cost, and longer duration of action^1–3^. Therefore, METH use and abuse continues to be a significant public health problem in the United States and all over the world^1–3^.

METH is a lipophilic compound that crosses the blood–brain barrier easily to cause long lasting effects in the brain^4,5^. Since METH’s chemical structure is similar to monoamines, it targets both dopaminergic (DA) and serotonergic (5HT) neurons in the brain. METH enters these neurons by binding to the membrane-bound DA transporter (DAT) and/or 5HT transporter^3^. There is also evidence that METH can cross neuronal membranes by passive diffusion mechanism^6^. Once inside the neuron, METH can alter the function of the vesicular monoamine transporter 2 (VMAT-2) and affect cytoplasmic monoamine concentrations and DA release^6^. METH administration is also known to increase DAT internalization and affect 5HT transporter functionality^6–8^. In addition, METH prevents degradation of neurotransmitters, reduces neurotransmitter reuptake, and causes an efflux of DAT and 5HT transporters^6–8^. These biochemical and cellular effects result in an increased neurotransmitter availability in the synapse^6–8^ and cause continuous stimulation of neurons to manifest the euphoric effects among METH users^9–11^.

In addition to its effects on DA and 5HT neurons, METH has been shown to affect glutamate neurotransmission^12,13^. There is evidence that METH’s effect on glutamate neurotransmission is mediated by increasing levels of extracellular glutamate^13,14^. However, the mechanism of glutamate homeostasis during METH exposure has not been clearly delineated. Glutamate is the principal excitatory neurotransmitter and is ubiquitously distributed in the brain^15–17^. Due to its critical role in neuronal plasticity, glutamate is involved in a number of critical brain functions^15–17^ including learning and memory^18^. It is important to note that glutamate is produced in the nerve terminals predominantly from two sources: (1) the tricarboxylic acid (TCA) cycle, and (2) glutamine produced by the glial cells^19,20^. Glutamate produced in the nerve terminals is packaged into vesicles by the vesicular glutamate transporters (vGLUT) and is released to the extracellular space upon stimulus of an action potential^20^. After release, the glial cells clear glutamate from the synapse via the glutamate transporters, GLAST and GLT-1, to maintain sub-neurotoxic levels of extracellular glutamate^21–23^. Maintaining glutamate homeostasis is critical for proper neuronal function since excessive glutamate has been implicated in neuronal excitoxicity^24^.

**Figure 1:**
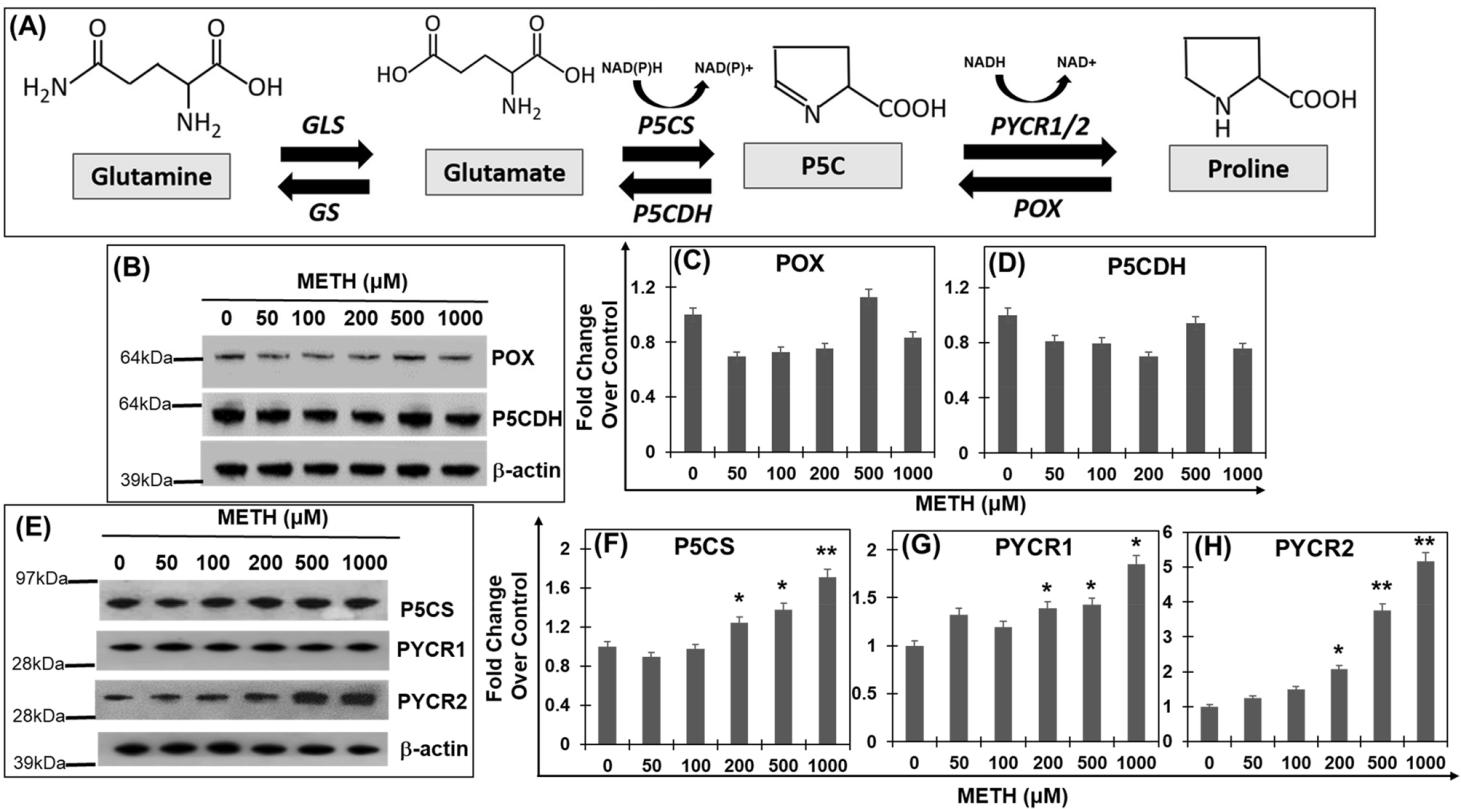
Acute METH exposure upregulates enzymes of proline synthetic pathway from glutamate. (**A**) Schematic depicting the metabolic link between glutamate and proline. Synthesis of proline begins with the conversion of glutamine to glutamate by the enzyme glutaminase (GLS). Glutamate is then converted by P5C synthase (P5CS) to glutamic-γ-semialdehyde that spontaneously cycles to Δ^1^-pyrroline-5-carboxylate (P5C). P5C is subsequently catalyzed to l-proline by P5C-reductase (PYCR). Conversely, catabolism of proline to glutamate is catalyzed in two consecutive steps by proline oxidase (POX) and P5C dehydrogenase (P5CDH). Glutamate is finally converted to glutamine by glutamine synthase (GS). (**B**–**D**) Effect of acute METH treatment on proline catabolic enzymes. SH-SY5Y cells were treated with increasing concentrations of METH for 24 h. Post-treatment cells were harvested and lysed. Cell lysates were subjected to immunoblot analyses. (**B**) Representative immunoblot of enzymes involved in catabolism of proline to glutamate (n = 3) (Fig. S1). (**C**,**D**) Densitometry analysis of POX and P5CDH, respectively normalized to β-actin. (**E**–**H**) Effect of acute METH treatment on proline metabolic enzymes. (**E**) Representative immunoblot of enzymes, P5CS, PYCR1 and PYCR2, which are essential for proline biosynthesis from glutamate (n = 3). (**F**–**H**) Densitometry analysis of P5CS, PYCR1, and PYCR2, respectively normalized to β-actin. Data presented in panels (**C**,**D**,**F**–**H**) are mean values of three independent experiments with error bars representing SEM. *p < 0.05, **p < 0.005 represent statistical comparison of untreated vs METH-treated cells.

The link between glutamate metabolism and carbohydrate/amino acid metabolism has been well established. Specifically, glutamate is channeled to the TCA cycle—a common pathway in carbohydrate and amino acid metabolism^25^. However, the metabolic link between glutamate and proline^26,27^ remains largely understudied. Proline, unlike other amino acids, has its α-amino group within a pyrrolidine ring, and thus it is the sole proteinogenic secondary (imino) amino acid^27^. Proline is also unique since it has its own set of metabolic enzymes^28^. It is known that glutamate can be converted to proline through Δ1-pyrroline-5-carboxylate (P5C) by the enzymatic activity of P5C synthase (P5CS) and P5C reductase (PYCR)^26,29^ (Fig. 1A). Conversely, proline can also be converted to glutamate through the catabolic pathway catalyzed by proline oxidase/dehydrogenase (POX/ PRODH) and P5C dehydrogenase (P5CDH)^25,26,29^. Given this important metabolic link, proline metabolism has been reported to play key roles in normal brain function and various neurological disorders.

Proline is abundantly found in the central nervous system (CNS)^30^. The presence of high affinity proline transporters has also been reported in a subset of glutamatergic neurons in rodent brain^31^. In animal models, elevated proline levels affect glutamatergic transmission^32^, depolarize neurons^33,34^, increase synaptic activity^35^, alter cognitive tasks, sensorimotor gating, and locomotor activity^36,37^. In humans, hyperprolinemia—a condition associated with abnormally elevated levels of proline has been linked to epilepsy, seizures and impaired cognitive function^38^. Hyperprolinemia is also linked to schizoaffective disorders^39^ and schizophrenia^40,41^. Even though these studies and others demonstrate key roles of proline in brain function and neurological disorders, the role of proline metabolism during drug exposure remains largely unknown.

In this study, we report a functional link between proline and glutamate metabolism during acute METH exposure. First, we utilized the SH-SY5Y neuronal cell model, that has been widely used to study neuronal biology^42,43^. SH-SY5Y cells are positive for tyrosine hydroxylase (TH) and dopamine-β-hydroxylase characteristic of catecholaminergic neurons that are known to be affected by METH^44^. In this model, we tested the functional contribution of proline metabolism in METH-exposure mediated alterations in glutamate levels. Interestingly, acute treatment with METH markedly induced the enzymes of the proline biosynthetic pathway. Specifically, a marked induction in the expression of P5CS and PYCRs was observed in METH treated neuronal cells. This induction was accompanied by a concomitant increase in intracellular proline levels. Surprisingly, METH exposure neither increased extracellular glutamate levels or caused neuronal excitotoxicity. However, blocking the proline synthetic arm by knocking-out P5CS increased both intracellular and extracellular glutamate levels in METH treated cells. Furthermore, treatment of proline auxotrophic-Chinese hamster ovary (CHO-K1) cells with METH significantly enhanced extracellular glutamate levels. These results suggested a functional link between biosynthesis of proline and glutamate during METH exposure. Finally, levels of proline biosynthetic enzymes, specifically, P5CS and PYCR2 were also significantly induced in the cortex of animals administered with METH and in the slices of cortical brain tissues exposed to METH. Collectively, our studies demonstrate activation of proline biosynthesis to sequester increased glutamate during acute METH exposure and imply an essential role of proline metabolism in limiting neuronal glutamate excitotoxicity.

## Results

### Acute METH exposure activates the proline synthetic pathway

METH exposure affects glutamate neurotransmission primarily by altering glutamate levels^12,13^. While, glutamine serves as the major source of glutamate^21–23^, proline metabolism is also closely linked to glutamate^25^ (Fig. 1A). However, the functional link between proline and glutamate during drug exposure induced glutamate neurotransmission is poorly understood. Therefore, we examined the effects of METH exposure on proline metabolism using the SH-SY5Y neuronal cell model. First, we measured the levels of enzymes of the proline catabolic arm- POX and P5CDH- which sequentially convert proline to P5C and then to glutamate (Fig. 1A). We treated SH-SY5Y cells with METH in a dose dependent manner using concentrations of 1 mM and lower. Although higher concentrations of METH have been used in published studies^45,46^, we chose concentrations below 1 mM since higher levels of the drug are rarely achieved in METH associated disorders^47,48^. Lysates of METH-treated and control cells were analyzed by immunoblot to measure the levels of POX and P5CDH. These analysis revealed that SH-SY5Y cells endogenously express all the key metabolic enzymes of proline (Fig. 1B,D). Interestingly, METH exposure minimally altered the expression of POX and P5CDH when compared to the untreated controls (Fig. 1B–D). Even at 1 mM concentration of METH, expression of these two enzymes were not significantly altered (Fig. 1B–D), suggesting that acute METH exposure minimally alters the proline catabolic arm that converts proline to glutamate (Fig. 1A).

We next measured the expression of enzymes of the proline biosynthetic arm (Fig. 1A), P5C synthase (P5CS) and P5C reductase (PYCR) that convert glutamate to proline by sequential catalysis^26^. Notably, the expression of P5CS was significantly increased in cells exposed to METH (Fig. 1E,F). Densitometry analysis illustrated a dose-dependent increase in P5CS expression in cells treated with METH relative to untreated cells (Fig. 1F). Then, we measured the levels of PYCRs- PYCR1, and PYCR2, which are involved in biosynthesis of proline (Fig. 1A)^27,29,49^. As presented in Fig. 1E,G,H, expression of the PYCR1 and PYCR2 was significantly increased in the METH treated cells. While the expression of PYCR1 was increased ~ 1.7-fold, the levels of PYCR2 was ~ sixfold higher in cells treated with 1 mM METH compared to the untreated control cells (Fig. 1E,G,H). Collectively, these results demonstrated that acute METH exposure activates the proline synthetic arm without altering the catabolic arm.

### METH exposure upregulates key proline metabolic enzymes in differentiated neuronal cells and cortical brain slices

**Figure 2:**
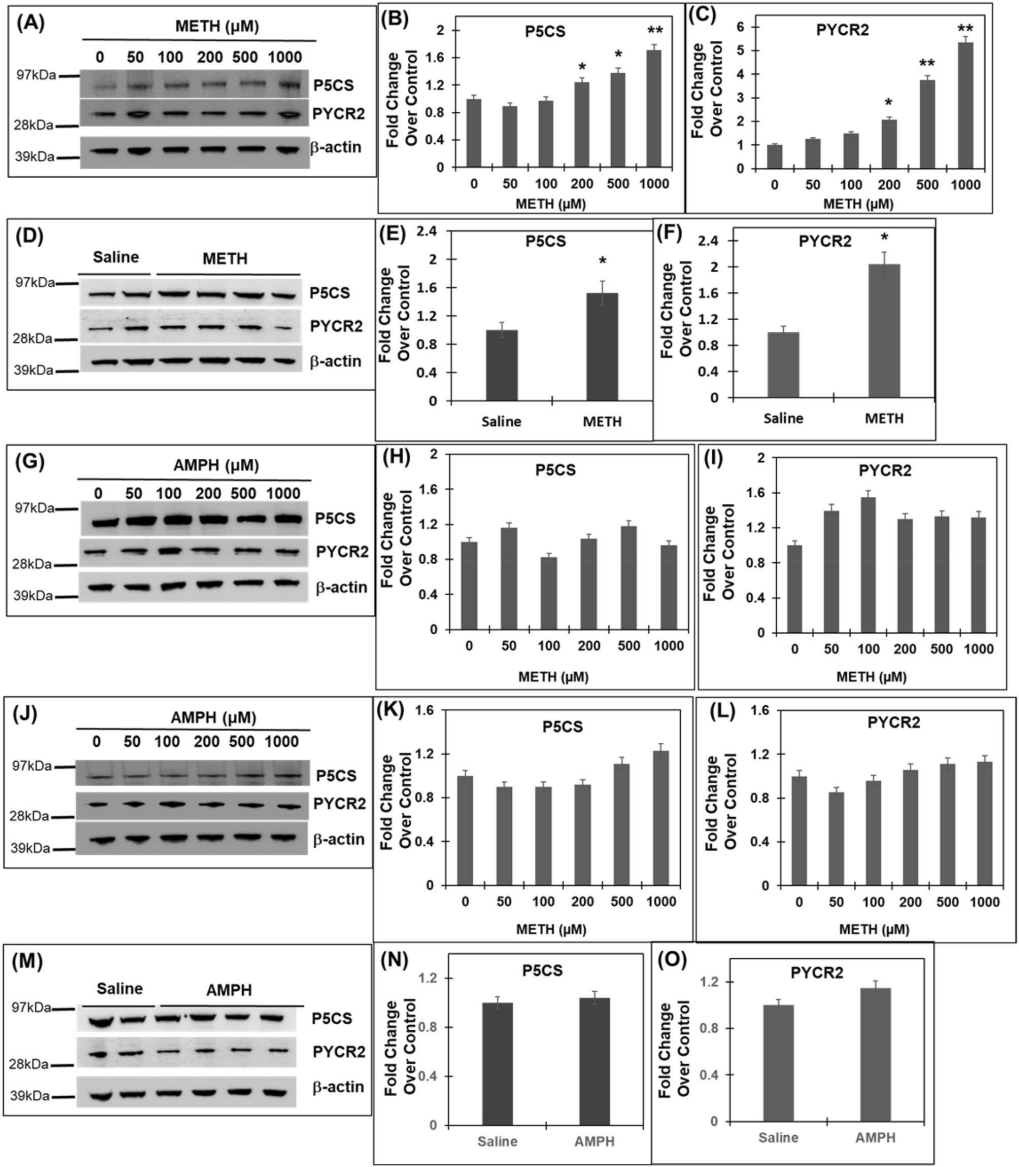
METH exposure upregulates P5CS and PYCR2 in differentiated neuronal cells and slices of cortical brain. (**A**–**C**) Differentiated SH-SY5Y cells were treated with increasing concentrations of METH for 24 h. Post-treatment cellular lysates were subjected to immunoblot analyses. (**A**) Representative immunoblot of P5CS and PYCR2 (n = 3). (**B**,**C**) Densitometry analysis of P5CS and PYCR2, respectively normalized to β-actin. (**D**–**F**) Slices of the frontal cortex were prepared from whole brain and were incubated in 200 µM METH (n = 4) or vehicle (n = 2). Tissue lysates were subjected to western blot analysis. (**D**) Representative immunoblot of P5CS and PYCR2 (n = 3). (**E**,**F**) Densitometry analysis of P5CS and PYCR2, respectively normalized to β-actin. (**G**–**I**) SH-SY5Y cells were treated with increasing concentrations of AMPH for 24 h. After treatment, cellular lysates were analyzed by immunoblot analyses. (**G**) Representative immunoblot of P5CS and PYCR2 (n = 3). (**H**,**I**) Densitometry analysis of P5CS and PYCR2, respectively normalized to β-actin. (**J**–**L**) Differentiated SH-SY5Y cells were treated with increasing concentrations of AMPH for 24 h and the cellular lysates were analyzed by immunoblot. (**J**) Representative immunoblot of P5CS and PYCR2 (n = 3). (**K**,**L**) Densitometry analysis of P5CS and PYCR2, respectively normalized to β-actin. (**M**–**O**) Brain slices of the frontal cortex were prepared from whole brain and were incubated in 200 µM AMPH (n = 4) or vehicle (n = 2). Lysates of the cortical tissues were analyzed by immunoblot. (**M**) Representative immunoblot of P5CS and PYCR2 (n = 3). (**N**–**O**) Densitometry analysis of P5CS and PYCR2, respectively normalized to β-actin. Data in panels (**B**,**C**,**E**,**F**,**H**,**I**,**K**,**L**,**N**,**O**) are presented as the mean ± SEM of at least three independent experiments. *p < 0.05, **p < 0.005 represent statistical comparison of untreated/saline-treated vs METH-treatment.

Our results in Fig. 1 showed upregulation of proline synthetic enzymes by METH exposure in undifferentiated SH-SY5Y cells. Even though the SH-SY5Y neuroblastoma cells show some characteristics of neurons, we have previously shown that these cells acquire a neuronal phenotype by differentiation utilizing ATRA^50^. Therefore, we used differentiated SH-SY5Y cells and treated them with METH in a dose dependent manner. 24 h post treatment cellular extracts were prepared to measure the expression of P5CS and PYCR2, as representatives of proline synthetic enzymes. Immunoblot analysis showed that METH treatment resulted in a significant upregulation of both P5CS and PYCR2 when compared to untreated controls (Fig. 2A–C). These observations are similar to the results obtained with the undifferentiated cells (Fig. 1). To further probe the physiological relevance of these cell-based observations, we carried out METH exposure experiments using slices of cortical regions of rodent brain. Given the technical challenges associated with brain slice experiments, 200 µM METH was selected based on the significant upregulation of both P5CS and PYCR2 in neuronal cells at this concentration (Figs. 1E–H, 2A–C). Brain slices were incubated in METH or vehicle (saline) for a 6-h period. Then cortical enriched tissue was isolated and immunoblot analysis was carried out of the tissue lysates. Data from these studies illustrated that P5CS and PYCR2 levels were significantly upregulated in the cortical brain slices upon METH exposure (Fig. 2D–F). Taken together, results in Figs. 1 and 2A–F establish that acute METH exposure upregulates the proline metabolic enzymes in neuronal cells and cortical brain slices.

Next, we determined whether activation of proline synthetic arm is specific to METH exposure or a general mechanism utilized by other ALS. To test this, we first treated undifferentiated SHSY-5Y cells with increasing concentration of Amphetamine (AMPH). Immunoblot analysis of cellular lysates showed a minimal but non-significant increase in P5CS and PYCR2 protein expression by AMPH exposure when compared to untreated controls (Fig. 2G–I). Interestingly, AMPH exposure of differentiated SH-SY5Y cells illustrated a modest increase in the expression of P5CS at 1000 µM METH. However, PYCR2 expression was minimally changed in the differentiated cells exposed to increasing concentrations of AMPH (Fig. 2J–L). Finally, exposure of brain slices to AMPH (200 µM) showed a lack of significant increase in the expression of both P5CS and PYCR2 (Fig. 2L–O). Collectively, results described in Fig. 2 strongly suggested that acute METH exposure activates the proline catabolic arm, whereas AMPH exposure lacks such activation.

### Acute METH exposure enhances proline levels but does not increase glutamate levels

**Figure 3:**
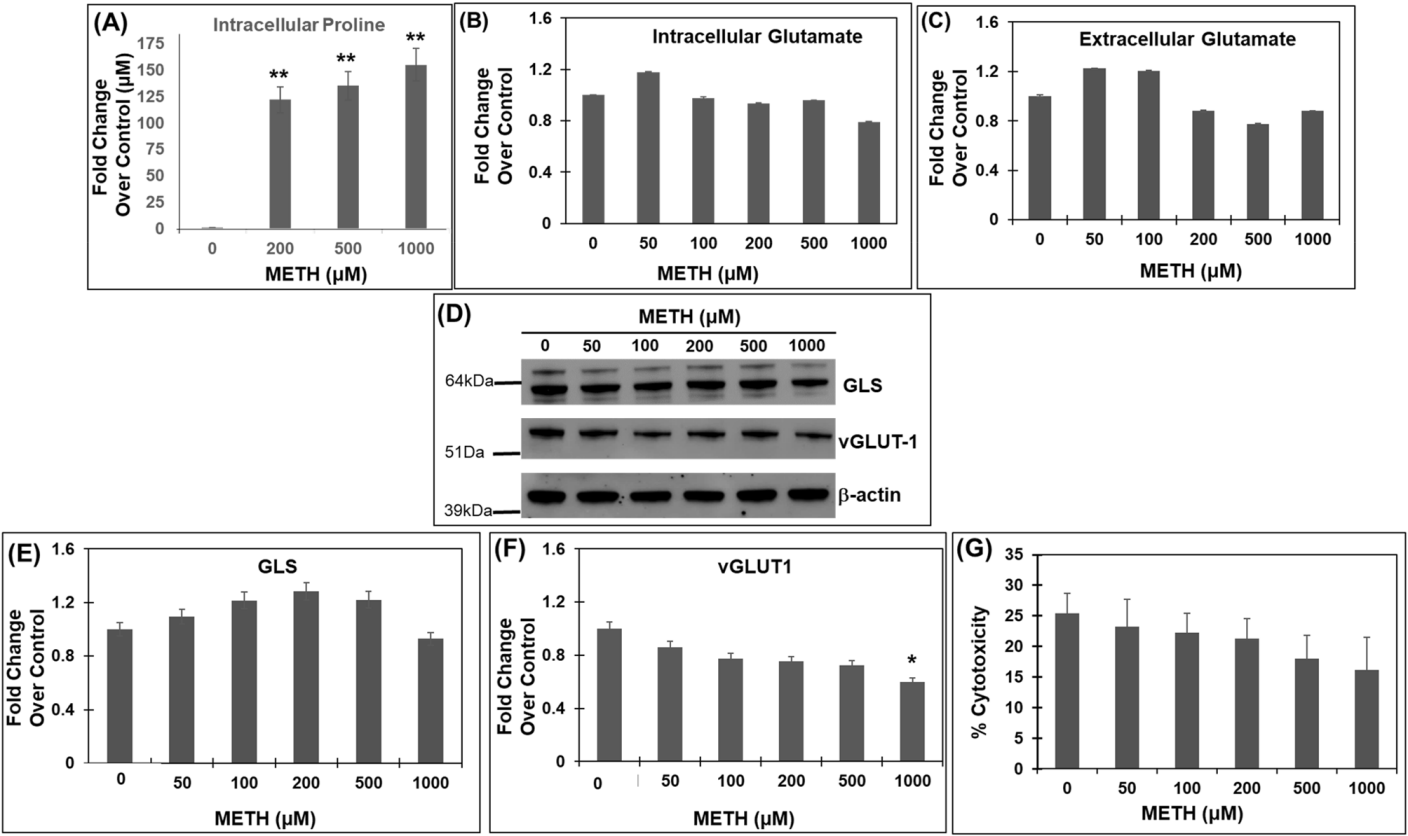
Acute METH exposure increases levels of proline without increasing glutamate levels. (**A**) Effect of acute METH treatment on proline levels-SH-SY5Y cells were treated acutely with METH at concentrations 200, 500, and 1000 µM. After treatment, the cells were harvested and the intracellular proline levels were measured by the acid-ninhydrin assay, that specifically detects proline. (**B**,**C**) Effect of Acute METH treatment on glutamate levels. SH-SY5Y cells were treated with increasing concentrations of METH for 24 h. Post-treatment cells were centrifuged and the cell extracts were used to measure intracellular glutamate while the cell-free supernatants were used to measure extracellular glutamate. Data are plotted as fold change in glutamate levels in METH-treated cells compared to control cells. (**D**–**F**) Effects of acute METH treatment on GLS and vGLUT1 expression—SH-SY5Y cells were treated with varying concentrations of METH under acute conditions. Cells were then harvested and cellular lysates were subjected to immunoblot analyses. (**D**) Representative immunoblot of GLS and vGLUT1 expression (n = 3). Densitometry analysis of GLS-1 expression in (**E**) and vGLUT1 expression in (**F**) normalized to β-actin. (**G**) Acute METH treatment does not induce cytotoxicity—SH-SY5Y cells were treated with increasing concentrations of METH for 24 h, following which the cells were centrifuged and culture supernatant were collected. Cytotoxicity was measured by LDH release assay (n = 3). Data presented in (**A**–**C**,**E**–**G**) are mean values of (n = 3) independent experiments conducted in triplicates with error bars representing SEM. **p < 0.005 represents statistical comparison of untreated vs METH-treated cells.

Results in Figs. 1 and 2 demonstrated that METH exposure upregulated the expression of P5CS and PYCR2. Since these enzymes catalyze the synthesis of proline from glutamate, we measured intracellular proline levels in METH treated SH-SY5Y cells. We chose 200, 500 and 1000 μM of METH for this experiment since both P5CS and PYCR2 were significantly upregulated at these drug concentrations (Figs. 1, 2). To quantify proline levels, we utilized the acid-ninhydrin assay that specifically detects proline^51,52^. Results from this assay illustrate that the levels of intracellular proline were significantly elevated in METH treated cells when compared to the untreated control cells (Fig. 3A). Specifically, treatment of cells with 200 μM METH resulted in a ~ 100 μM increase in proline levels relative to the untreated cells. Similarly, treatment with 500 μM METH further increased the proline levels up to ~ 130 μM and 1000 μM METH enhanced proline levels up to ~ 155 μM. These results indicate that acute METH exposure significantly enhances proline biosynthesis in accordance with the induction of proline synthetic enzymes.

A number of studies have shown that METH exposure alters glutamate levels^53–55^. Therefore, we tested whether the increased proline levels were due to higher glutamate in METH-treated cells. We measured the levels of both intracellular and extracellular glutamate in SH-SY5Y cells. We treated these cells with 50–1000 µM METH for 24 h. Following that, we measured glutamate levels in the cellular lysates and supernatants by a colorimetric assay. As shown in Fig. 3B,C, treatment of SH-SY5Y cells with METH at concentrations of 0–1000 µM did not significantly increase either intracellular (Fig. 3B) or extracellular (Fig. 3C) glutamate levels. Surprisingly, there was a slight decrease in both intracellular and extracellular glutamate levels (~ 20–30%) in cells treated with METH at the concentrations 200–1000 µM. Collectively these results suggest that acute METH treatment at concentrations ≤ 1 mM minimally affects intracellular and extracellular glutamate levels.

A lack of increased glutamate in METH treated cells could be due to reduced glutamate synthesis from glutamine-the major source of glutamate in neurons (19,20). To test this hypothesis, we measured the levels of glutaminase (GLS) enzyme that converts glutamine to glutamate in neurons (22–24). Western blot analysis revealed that METH treatment did not reduce GLS levels in SH-SY5Y cells, (Fig. 3D,E). Interestingly, a modest increase in GLS expression was observed in METH treated cells (Fig. 2D,E). These observations suggested that glutamate synthesis is not reduced in METH treated cells. Thus, a lack of increased glutamate during acute METH exposure was most likely not due to reduced synthesis from glutamine.

Extracellular glutamate levels are also dependent on packaging of intracellular glutamate by the vesicular glutamate transporters: vGLUT1, vGLUT2, and vGLUT3 (22–24). Among these transporters, the functional characteristics of vGLUT1 and vGLUT2 for glutamate transport is similar^56^. Interestingly, vGLUT3 has been reported to be found in cholinergic neurons but not in glutamatergic cell populations^57^. Therefore, we measured the levels of vGLUT1 as the selected marker of glutamate release in METH treated cells. Surprisingly, western blot analysis revealed a dose-dependent decrease in vGLUT1 expression with increasing concentration of METH (Fig. 3D,F). The reduction in vGLUT1 expression suggested that the packaging of glutamate was most likely reduced in METH treated cells. Interestingly, the reduction in vGLUT1 expression is correlated with the decrease in extracellular glutamate levels in cells treated with METH at concentrations of 200–1000 µM (Fig. 3C). Finally, we also measured neuronal cytotoxicity by LDH assay in the supernatants of METH-treated cells. METH treatment did not cause any cytotoxicity even at 1 mM concentration when compared to untreated control cells (Fig. 3G). Collectively, these results demonstrate that acute METH exposure at concentrations below 1 mM significantly enhances proline levels but minimally increases extracellular glutamate and lack the ability to cause neuronal cytotoxicity.

### P5CS is the key enzyme that regulates glutamate levels during acute METH exposure

**Figure 4:**
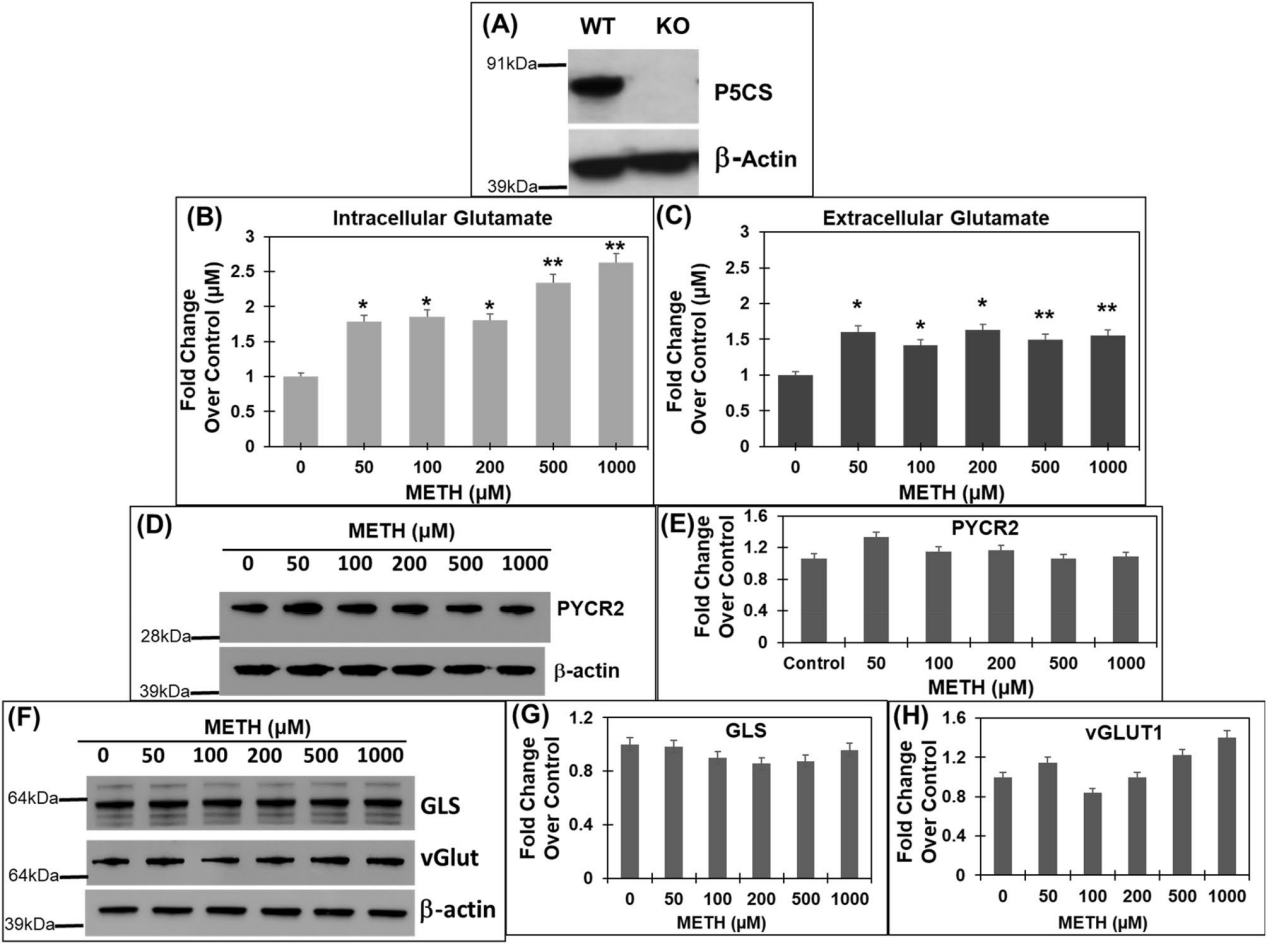
METH treatment of P5CS knockout (*P5CS*-KO) SH-SY5Y cells significantly increases glutamate levels. The *P5CS-*KO SH-SY5Y cells were generated from wild-type SH-SY5Y cells using CRISPR/Cas9 system. Following which the *P5CS*-KO cells were treated with METH for 24 h and cellular lysates and cell-free supernatants were collected. (**A**) Western blot showing a lack of P5CS expression in *P5CS*-KO vs. WT SH-SY5Y cells (n = 3). β-actin was used as a loading control. (**B**,**C**) Effect of METH on glutamate in *P5CS*-KO cells. (**B**) The cell extracts were used to measure intracellular glutamate while (**C**) the cell-free supernatants were used to measure extracellular glutamate (n = 3). A marked increase in levels of both intracellular and extracellular glutamate was obtained in *P5CS*-KO cells after METH treatment. (**D,E**) Effect of acute METH treatment on PYCR2 in *P5CS*-KO cells. Representative western blot of PYCR2 in (**D**) following acute METH treatment (n = 3). β-actin was used as a loading control. (**E**) Densitometric analyses of PYCR2 normalized to β-actin. (**F**–**H**) Effect of acute METH treatment on GLS and vGlut1 in *P5CS*-KO cells. (**F**) Representative western blot of GLS and vGLUT1 in METH-treated *P5CS*-KO SH-SY5Y cells (n = 3). β-actin was used as a loading control. (**G**,**H**) Densitometric analyses of GLS and vGLUT1 western blot normalized to β-actin. Data are presented as the mean ± SEM of at least three independent experiments. *p < 0.05, **p < 0.005 represents statistical comparison of untreated vs METH-treated cells.

Our results in Fig. 3A demonstrated that acute METH exposure activates proline biosynthesis. However, a lack of alterations in glutamate levels in these cells (Fig. 3B,C), led us to hypothesize that activation of proline biosynthesis may aid in regulating glutamate levels during acute METH exposure. To test this, we created SH-SY5Y cells that are deficient in proline biosynthesis from glutamate. We specifically depleted the P5CS enzyme since synthesis of proline from glutamate is regulated by P5CS (Fig. 1A)^26^ and METH significantly increased P5CS levels (Figs. 1, 2). Even though PYCR2 was also upregulated by METH, blocking the reductase step required depletion of both PYCR1 and PYCR2 due to the functional redundancy of these two enzymes. Therefore, we generated *P5CS* knockout SH-SY5Y cells (*P5CS-*KO*)* using CRISPR/Cas9 genome-editing approach^58^. We employed a lentiviral vector-based expression of nuclease-active Cas9 and *P5CS* gRNAs and selected single clones of *P5CS*-KO. Western blot analysis confirmed lack of P5CS expression in the *P5CS-*KO cells when compared to the wild type cells (Fig. 4A). We then treated the *P5CS*-KO cells with METH in a dose-dependent manner for 24 h. Measurement of glutamate levels showed a significant increase in intracellular glutamate levels in the *P5CS*-KO cells upon METH treatment (Fig. 4B). For instance, METH exposure at 50 µM resulted in ~ 1.8-fold increase in intracellular glutamate levels in *P5CS*-KO cells compared to the untreated control. This increase was further enhanced to ~ 2.6-fold with 1 mM METH treatment *P5CS*-KO cells (Fig. 4B). Interestingly, acute METH exposure also significantly elevated extracellular glutamate levels in the *P5CS*-KO cells (Fig. 4C). These results are in contrast to the minimal effect of METH exposure on glutamate levels in the wild type cells that express P5CS (Fig. 3B,C).

To further solidify the role of P5CS in regulating glutamate levels, we probed the expression of PYCR2 in the *P5CS*-KO cells, since METH treatment increased PYCR2 expression in wild type cells (Figs. 1, 2). Results presented in Fig. 4D,E clearly show that METH exposure minimally alters the expression of PYCR2 in the *P5CS*-KO cells. Moreover, in *P5CS*-KO cells treatment of METH minimally affected GLS expression (Fig. 4F,G), suggesting that depletion of P5CS does not affect glutamate synthesis from glutamine. Interestingly, a marginal increase in vGLUT1 expression was observed in the *P5CS*-KO cells upon METH treatment above 500 µM (Fig. 4H). This increase is consistent with the increased glutamate levels observed in the *P5CS*-KO cells after METH exposure. Collectively, these results establish that depletion of P5CS causes accumulation of glutamate and strongly suggest the involvement of proline biosynthetic arm in maintaining METH-induced glutamate homeostasis.

### METH exposure increases glutamate levels in proline-auxotrophic Chinese hamster ovary (CHO) cell line

**Figure 5:**
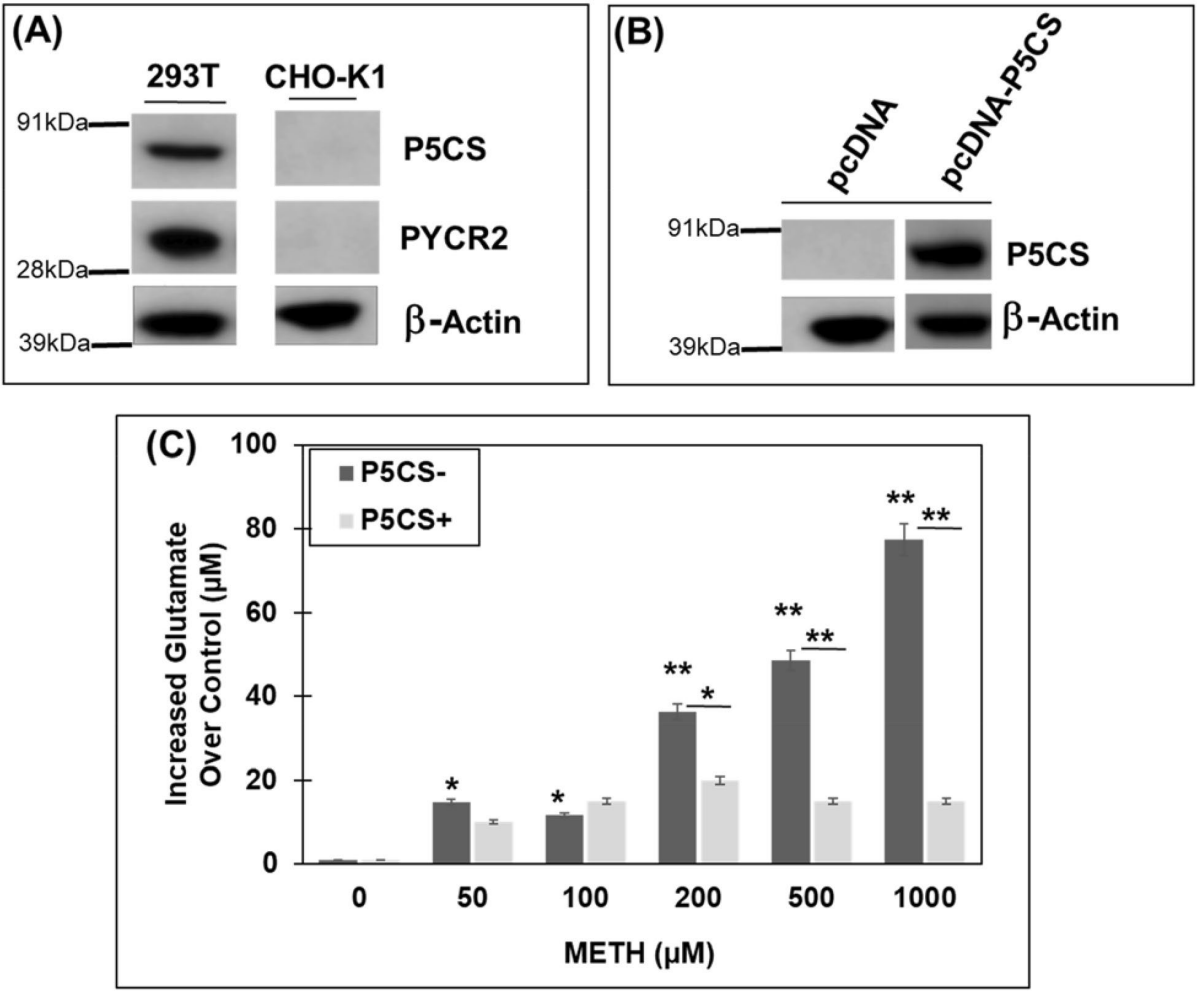
METH treatment increases glutamate levels in proline-auxotrophic (CHO-K1) cells. CHO-K1 cells are proline auxotrophs that lack P5CS and PYCR2. (**A**) Western blot showing the absence of P5CS and PYCR2 in CHO-K1 cells (n = 3). β-actin was used as loading control. HEK293T cells that express both P5CS and PYCR2 was used as positive control. (**B**) Overexpression of P5CS in CHO-K1 cells. An expression construct of P5CS was generated using pcDNA 3.1. CHO-K1 cells were transfected with either pcDNA-empty vector or pcDNA-*P5CS* and post-transfection western blot was performed to confirm the expression of P5CS (n = 3). β-actin was used as a loading control. (**C**) Effect of METH treatment on glutamate levels in CHO-K1 in the presence and absence of P5CS expression. CHO-K1 cells were transfected with either pcDNA-empty vector or pcDNA-*P5CS*. Post-transfection the cells were treated with varying concentrations of METH under acute conditions. Glutamate levels were measured in the cell-free supernatants after 24 h (n = 3). Data represent the increase in glutamate levels over control untreated cells and are the mean ± SEM of at least three independent experiments. In (**C**), *p < 0.05, **p < 0.005 represents statistical comparison of untreated vs METH-treated cells shown in black bars, whereas *p < 0.05, **p < 0.005 represents comparison of METH-treated cells in the absence and presence of P5CS shown in gray bars.

Our results demonstrated that P5CS expression is associated with alterations in glutamate levels during acute METH exposure (Fig. 4). To further probe a functional link between proline synthetic arm and glutamate, we exploited the proline auxotrophic nature of Chinese Hamster Ovary (CHO) cells. CHO cells lack the proline biosynthetic arm that is needed to catalyze proline from glutamate^59,60^. Specifically, these cells do not express P5CS and PYCR2 as measured by western blot analysis (Fig. 5A). Therefore, to further strengthen the role of P5CS during glutamate homeostasis, CHO cells were treated with increasing doses of METH for 24 h. Then extracellular glutamate levels were measured in the culture supernatants of treated and untreated cells. As shown in Fig. 5C, a dose-dependent increase in glutamate levels was detected in the presence of increasing concentrations of METH.

Next, we tested whether the higher levels of glutamate in METH-treated CHO cells is a consequence of lack of proline synthesis from glutamate. We exogenously expressed P5CS in the CHO cells by transfecting a P5CS expression construct. Post-transfection, western blot analysis of the cellular lysates of these cells confirmed the expression of P5CS compared to the lack of P5CS expression in wild type cells (Fig. 5B). Then, the P5CS-expressing CHO cells were exposed to increasing concentrations of METH for 24 h and extracellular glutamate levels were measured in the supernatants. Results from these assays show that P5CS expression significantly abrogates the increase in extracellular glutamate upon METH exposure as compared to the P5CS-lacking wild type cells (Fig. 5C). Taken together, these observations provide strong evidence that P5CS regulates glutamate levels during acute METH exposure.

### Acute METH exposure activates proline biosynthetic pathway in the cortical regions of mice brain

**Figure 6:**
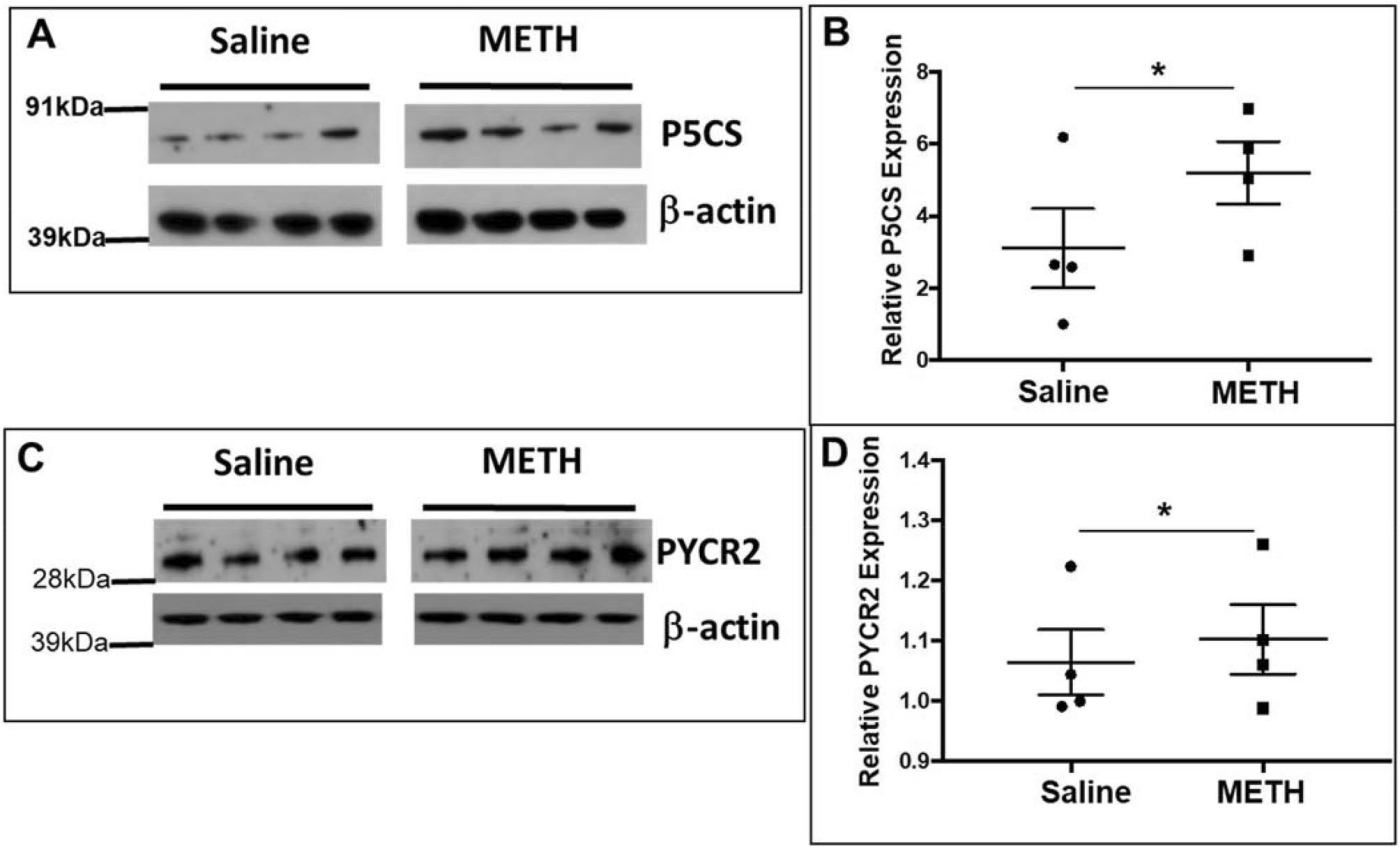
METH treatment activates proline synthetic pathway in cortical regions of mice brain-Mice were injected intra-peritoneally (IP) with a single dose of METH (2 mg/kg) for acute METH treatment. Then the animals were euthanized after 24 h and whole brain was isolated from METH treated (n = 4) and saline-treated (n = 4) animals. Cortical regions were then sliced and homogenized for measuring the levels of proline synthetic enzymes. (**A**) Representative western blot probing P5CS levels in the cortices of saline treated mice and METH treated mice with β-actin used as a loading control (n = 3). (**B**) Quantitative representation of P5CS western blot of either saline or METH treated mice. (**C**) Western blot probing for PYCR2 in the cortical region of saline treated mice and METH treated mice with β-actin used as a loading control (n = 3). (**D**), Quantitative representation of PYCR2 western blot of either saline or METH treated mice. Data are presented as the mean ± SEM of at least three independent experiments. *p < 0.05 represents statistical comparison of untreated vs METH-treated samples.

Our studies using neuronal cell model and proline auxotrophic cells (Figs. 1, 2, 3, 4, 5) demonstrated a key role of proline biosynthetic pathway in regulating glutamate levels during acute METH exposure. To determine the in vivo significance of these observations, we investigated the effects of METH exposure on the proline synthetic pathway in a rodent model. Mice were administered with either METH (2 mg/kg IP) or saline under acute conditions for 24 h. Then, the cortical regions of the brain were isolated and total protein from these brain tissues were extracted and subjected to western blot analyses. We focused on the cortical regions, since glutamatergic neuronal projections originating in the prefrontal cortex (PFC) extend to the striatum, nucleus accumbens (NAc), ventral tegmental area (VTA) and substantia nigra of the midbrain^61,62^ and METH has been shown to affect glutamate in the PFC of mice brain^63^. Data in Fig. 6A show that P5CS is constitutively expressed in the cortical region of mouse brain. Upon acute METH treatment of these mice, a significant increase in P5CS levels was detected when compared to the control animals, (Fig. 6A,B), suggesting upregulation of P5CS during METH exposure. Furthermore, protein levels of the PYCR2 were also increased in the cortical tissues of METH administered animals (Fig. 6C,D). However, the level of increase in PYCR2 was lower when compared to the significant increase in P5CS levels. These data are in accordance with the results obtained with the neuronal cell model (Figs. 1, 2). Collectively, these observations support the activation of the proline synthetic arm in the cortical region of mice brain during acute METH exposure.

## Discussion

Glutamate is the key excitatory neurotransmitter in the brain^15–17^. During glutamatergic neurotransmission, glutamate is packaged in presynaptic neurons and released into the synapse^15–17^. The released glutamate binds to ionotropic and metabotropic receptors on postsynaptic neurons to propagate the incoming signals^15–17^. Subsequently, high-affinity transporters present on the glial cells and neurons rapidly sequester the glutamate to facilitate a continuous cycle of neurotransmitter activity^64^. Given the central role of glutamate in neurotransmission, it is involved in most aspects of normal brain function including cognition, memory, and learning^18^. Glutamate also plays a critical role in drug reward, reinforcement, and relapse^65–68^. Importantly, recent studies suggest that in individuals with substance use disorder, glutamate is associated with synaptic plasticity^65–68^. Specifically, METH has been reported to affect glutamate neurotransmission by altering glutamate levels^14^. However, the mechanism by which METH exposure affects glutamate homeostasis is poorly understood.

METH has been shown to alter the extracellular concentrations of both glutamate and dopamine in the brain^13^. For example, chronic treatment with high doses of METH has been reported to increase glutamate efflux in the striatum^14^. Depletion of striatal dopamine levels with low dose METH has been shown to enhance glutamate release^69^. Similarly, a low dose of METH has been demonstrated to increase extracellular dopamine but not glutamate in PFC and striatum^63^. Additionally, repeated METH administrations were shown to enhance cortical glutamate efflux^63^. Interestingly, a single injection of a high dose of METH (30 mg/kg, i.p.), was reported to cause glutamate depletion^70^. These observations suggest that alterations in the levels of glutamate are dependent on the duration and dose of METH exposure. Accordingly, our results with a neuronal cell model demonstrated that acute METH exposure at concentrations below 1 mM minimally altered extracellular glutamate levels. It is noteworthy that majority of the published studies examined the effects of long-term exposure and relatively high doses of METH on glutamate^47,71^. Therefore, based on our results we speculate that a single dose of METH below 1 mM concentrations causes minimal changes in glutamate homeostasis.

Neuronal glutamate levels are regulated by a multi-prong mechanism that involve glutamate biosynthesis from glutamine and synaptic vesicle loading of intracellular glutamate. Glutamate synthesis is dependent on the glutamine–glutamate cycle between astrocytes and neurons^72^. In this metabolic cycle, GLS is primarily responsible for synthesizing glutamate from glutamine to continuously replenish the neurotransmitter pool (22–24). While, GLS regulates glutamate biosynthesis from glutamine (22–24), vGLUTs mediate the transport of glutamate into synaptic vesicles^73,74^ and vGLUT expression determines the amount of glutamate packaged into vesicles^73,74^. Specifically, in glutamatergic neurons the functionally similar vGLUT1 and vGLUT2 are responsible for glutamate transport^56^. Interestingly, our results demonstrated that expression of GLS remains mostly unaffected in METH treated cells (Fig. 3). Moreover, with acute METH treatment, a minimal to marginal reduction in vGLUT-1 expression was detected (Fig. 3). These observations strongly suggest that acute METH exposure minimally alters ongoing glutamate biosynthesis and vesicular packaging, consistent with the minimal effect of METH on glutamate levels under acute exposure conditions.

The glutamine–glutamate biosynthetic cycle is not stoichiometric, but is an open biochemical pathway wherein glutamate interfaces with several other metabolic pathways including carbohydrate and amino acid metabolism through the TCA cycle^70^. In this context, our results describing activation of proline synthetic arm by METH exposure highlights the novel role of proline biosynthesis during METH exposure. Glutamate can be channeled for proline synthesis through P5C. These biochemical reactions are sequentially catalyzed by P5CS and PYCR (Fig. 1A). Remarkably, our data showed that acute METH treatment resulted in a marked enhancement in both P5CS and PYCR2 expression (Figs. 1, 2). Concomitant increase in intracellular proline levels (Fig. 3A) indicated that induction of these two enzymes are most likely responsible for synthesizing proline from glutamate. Moreover, this increase was observed only with METH and not AMPH indicating the effect to be specifically mediated by METH. Previously, a genome-wide study, analyzed METH-induced mRNA expression in rodent brain to reveal the potential regulatory consequences in response to METH^75^. Pathways upregulated by METH included proline metabolism especially the proline synthetic enzyme P5CS (encoded by *ALDH18A1*) and PYCRs^75^. Similarly, a metabolomic study of brain tissues from METH affected animals uncovered neurochemical signatures related to the metabolism of amino acids including glutamate and proline^76^. Similar to METH, morphine is also known to affect glutamatergic neurotransmission^77,78^. In a proteomics analysis, PYCR2 was identified as one of the proteins that was induced by morphine in mouse hippocampal postsynaptic density-associated proteins (HPSD)^79^. These studies indicate an interplay between glutamate and proline metabolism. However, the functional relevance of proline metabolism during METH exposure is unknown. Therefore, our studies showing a functional association between glutamate and proline metabolism during METH exposure are highly significant.

Our results provide strong evidence of glutamate efflux to proline biosynthesis. Specifically, a marked induction in the expression of P5CS was observed during acute METH exposure not only in the SH-SY5Y neuronal cells but also in brain cortical region of mice. Interestingly, disruption in proline biosynthesis by knocking out of P5CS dramatically increased both intracellular and extracellular glutamate with METH treatment. The role of P5CS was further confirmed using a proline auxotrophic cell line (CHO-K1) that lacks the proline biosynthetic machinery. A marked enhancement in glutamate levels was also observed in these cells with METH treatment and this effect was abrogated by overexpression of P5CS. Collectively, these results demonstrated sequestration of neuronal glutamate pool towards proline biosynthesis. A lack of neuronal cell death by acute METH exposure even at 1 mM concentrations supports the hypothesis that sequestration of glutamate to proline is a critical mechanism activated to avert the accumulation of excess glutamate and prevent excitotoxicity.

Sequestration of glutamate to proline may have significant implications in the CNS. For instance, P5C formed during proline synthesis from glutamate can serve as the source for carbon exchange between the TCA and urea cycle^28^. Proline can also be deposited in collagen, the most abundant protein by weight in the human body^80^. Approximately, 25% of proline residues make up collagen, therefore, collagen has been suggested to be both a dump as well as a reservoir for proline^80^. This may be very relevant in the context of the extracellular matrix (ECM) of neurons. For example, excess glutamate can be converted to proline and deposited into the ECM as collagen without dramatically altering extracellular proline levels. High levels of proline have been shown to have excitotoxic properties and studies have shown that addition of proline to hippocampal slices decreased glutamate uptake causing glutamate to accumulate^81^. It is plausible that once the threshold for proline deposition is reached, especially with chronic METH, it may result in the accumulation of proline, which may also contribute to the glutamate excitotoxicity. Thus, the interplay between glutamate and proline may be an important regulator in METH-induced glutamate excitotoxicity.

**Figure 7:**
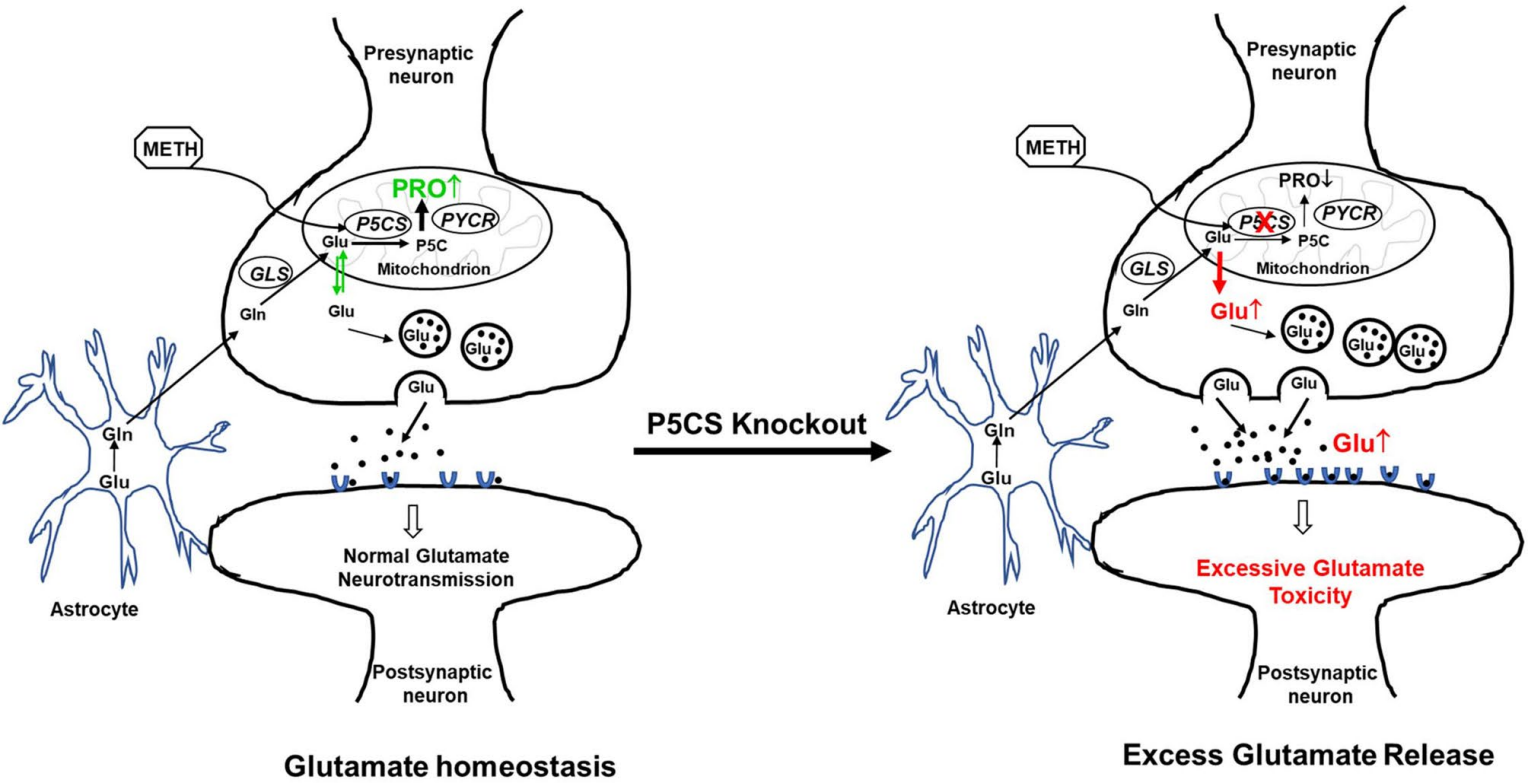
Proposed model demonstrating the key role of P5CS in maintaining glutamate homeostasis during acute METH exposure. The schematic model shows presynaptic and postsynaptic neurons along with astrocytes and highlights the pathways involved in glutamate homeostasis in the brain. The left panel depicts the sequestration of excess glutamate in the neuron for proline biosynthesis during acute METH exposure. Specifically, METH-induced enhancement of P5CS expression is associated with utilization of glutamate for proline synthesis, thus maintaining glutamate homeostasis. Whereas, the right panel describes that depletion of P5CS blocks the proline synthetic arm from glutamate and abrogates glutamate sequestration for proline biosynthesis. This metabolic disruption results in an increased levels of glutamate that could be detrimental for the neurons.

Importantly, we envision that increased conversion of glutamate to proline offers a bioenergetic metabolic advantage. The bioenergetic cost of maintaining homeostatic levels of synaptic glutamate is expensive^82^. Glucose via glycolysis provides the main source of energy for maintaining glutamate homeostasis in the brain. However, for efficient glycolysis a sustained supply of electron acceptor nicotinamide adenine dinucleotide (NAD^+^) is essential^83,84^. Proline biosynthesis has been shown to augment glycolysis by affecting the levels of NAD^+^, especially in cancer metabolism^85–87^. Therefore, our results demonstrating METH-induced proline biosynthesis may confer a metabolic bioenergetic advantage to neurons in response to increased neuronal stimulus by METH. In summary, our results identify a key role of P5CS during METH exposure and highlight that sequestering excess glutamate for proline biosynthesis is a key mechanism to maintain glutamate levels (Fig. 7).

## Materials and methods

All the methods used in this study are in accordance with relevant guidelines and regulations.

### Reagents

Methamphetamine hydrochloride (METH), Amphetamine hydrochloride (AMPH), all-trans-retinoic acid (ATRA) were obtained from Sigma-Aldrich Chemicals (USA). The primary antibodies used were as follows: anti-P5CS (cat# NBP1-83324) was purchased from Novus Biologicals (USA), anti-PYCR1 (cat# 13108-1-AP, anti-PYCR2 human (cat# 55060-1-AP, anti-PYCR2 mouse (cat #17146-1-AP, anti-P5CDH (cat# 11604-1-AP), anti-GLS (cat# 12855-1-AP) were purchased from Proteintech (USA), anti-POX IgG was a gift from Dr. James Phang (NCI-Frederick), anti-vGLUT1 (cat# 48-2400) was purchased from Thermo Fisher (USA), and anti-β-actin was obtained from Sigma-Aldrich Chemicals (USA). The secondary antibodies used were goat anti-rabbit or goat anti-mouse purchased from BioRad laboratories (USA).

### Cell culture and METH treatment

Human neuroblastoma cells (SH-SY5Y), Chinese hamster ovary (CHO-K1) cells were purchased from American Type Culture Collection (Manassas, VA). The SH-SY5Y cells were maintained in Dulbecco's modified Eagle’s medium (DMEM) (Gibco) supplemented with 10% fetal bovine serum (FBS) (Gibco), 2 mM Glutamine (Gibco), and 1000 U/mL penicillin, and 100 mg/mL streptomycin (Gibco). CHO-K1 cells were grown and maintained in Ham’s F-12K medium with 10% FBS, 2 mM Glutamine, 1000 U/mL penicillin, and 100 mg/mL streptomycin. SH-SY5Y were differentiated with ATRA as per published methods (48). All cells were maintained at 37 °C and 5% CO_2_ before and during treatments. The cells were treated acutely for 24 h with METH in a dose dependent manner at physiologically relevant concentrations of 50–1000 μM.

### Cloning

All three P5CS-targeting sgRNA sequences used in this study were from Doench et al*.*^88^ and each sgRNA was individually cloned into the TLCV2 plasmid vector (Addgene) by following the recommended protocol with modifications^58^. Briefly, the TLCV2 plasmid was first digested with BsmB1 by assembling a reaction mixture containing the plasmid DNA, Buffer 3.1 (NEB), and BsmB1 (NEB) and incubating at 55 °C for 30 min, then dephosphorylated by incubating the restriction digestion products with rSAP (NEB) at 37 °C for 30 min, and finally gel-purified. Each pair of oligos corresponding to a sgRNA was phosphorylated and annealed by assembling a reaction mixture containing the oligos, T4 ligation buffer (NEB), and T4 Polynucleotide Kinase (NEB) and incubating at 37 °C for 30 min followed by incubation at 95 °C for 5 min and then ramping down to 25 °C at 5 °C/min. The annealed oligos were diluted 200 fold and then ligated to the BsmB1-digested and dephosphorylated TLCV2 in a ligation mixture containing T4 ligation buffer (NEB) and T4 DNA ligase (NEB) and incubating at room temperature for 1 h. Subsequently, NEB-Stbl competent cells, prepared using the Mix&Go Transformation Kit (Zymo Research, USA) were transformed with an aliquot of the ligation reaction products as per manufacturer-recommended protocol and the bacterial recombinants were confirmed by Sanger sequencing of the isolated plasmids.

To construct the P5CS overexpression plasmid, the *P5CS* ORF was obtained by RT-PCR (abm, Canada) from SupT1 total RNA using P5CS-specific primers (forward: 5-TTA TAG GAT CCG CCA CCA TGT TGA GTC AAG TTT ACC-3′ and reverse: 5′-TGA CAC TCG AGT CAG TTG GTG TTT CTC TGA G-3′) containing BamH1 and Xho1 restriction enzyme sites. The BamH1 and Xho1 digested *P5CS* ORF amplicon was ligated to compatible ends of the pcDNA 3.1 plasmid (Addgene). The recombinant plasmids were confirmed by colony PCR and Sanger sequencing.

### Lentivirus transduction

HEK293T cells (5 × 10^5^) seeded per well in 6-well plates and cultured overnight were transfected with plasmid DNAs using the PEI transfection reagent (Polysciences Inc.). For transfecting cells in each well, a 4:3:1 ratio of lentiviral transfer plasmid (described above), packaging plasmid psPAX2, and pseudotyping envelope plasmid pMD2.G, and the PEI at a DNA:PEI ratio of 1:3 were used. After overnight culturing, the culture medium was removed and the cells were replenished with fresh medium. Forty-eight hours post transfection, the lentivirus-containing culture medium was collected, centrifuged at 500×*g* for 5 min at room temperature to remove cell debris, and the supernatant was filtered through 0.45 μm filter. Unused virus stocks were stored at − 80 °C for later use.

### P5CS CRISPR/Cas9 knockout in SH-SY5Y cells

For generation of stable cell lines, SH-SY5Y cells were inoculated with lentivirus for 24–48 h, then replenished with fresh culture medium containing the selection drug puromycin at 1 μg/mL. Cells were replenished with fresh culture medium containing puromycin every 2 days until majority of the uninfected control cells were eliminated (in ~ 7–10 days). To generate single cell clones, cells were treated with 1 μg/mL DOX for 24 h and the GFP positive cells were sorted out by flow cytometry. The GFP-positive single cells were then seeded into a 96-well plate and allowed to expand. The cell clones that were depleted of the target protein as analyzed by western blot were used in functional experiments.

### Lactate dehydrogenase (LDH) assay

To determine cytotoxicity, LDH release was measured with the LDH Cytotoxicity Assay Kit (Pierce) according to the manufacturer's protocol. 5 × 10^5^ SH-SY5Y cells were plated into 6-well plates and maintained overnight before treatment, following which the cells were treated with METH at various concentrations for 24 h. After treatment, the cell supernatants were collected by centrifugation. Supernatants were assayed for LDH in triplicates as per the manufacturer’s protocol. Absorbances were measured at 490 nm and 680 nm using a spectrophotometer (Biotek). The percentage of cytotoxicity was calculated based on the percentage difference compared with the LDH-positive control provided with the kit.

### Glutamate assay

5 × 10^5^ SH-SY5Y cells were plated in 6-well plate overnight and treated with METH for 24 h. Supernatants were collected, briefly centrifuged to remove cell debris, and analyzed by a Glutamate Colorimetric Assay kit purchased from Cell Biolabs as per the instructions from the manufacturer. For measuring intracellular glutamate, the cells were lysed by sonication in sucrose buffer. After lysis, the cell extracts were centrifuged at 15,000×*g* for 5 min and the supernatants were subjected to glutamate assay using the kit. All the reactions were performed in triplicates. The glutamate concentrations in the samples were determined by plotting the data against the respective standard curve that was generated in parallel using a series of known glutamate concentrations ranging from 6.25 to 400 µM.

### Measurement of intracellular proline levels

5 × 10^5^ SH-SY5Y cells were plated per well in 6-well plates overnight and treated with METH for 24 h. After treatment cells were harvested and lysed in cold PBS with 1% Triton X-100. The cellular debris were removed by centrifugation (10,000×*g*). The supernatants were transferred to a boiling water bath, and intracellular amino acids were extracted by boiling for 10 min. After centrifugation (5 min, 4 °C, 15,000×*g*), the supernatant was free of proteins, and intracellular proline was determined as described^51,52^. Briefly, 100 µL of the supernatant was incubated with 100 µl of acid-ninhydrin (0.25 g ninhydrin dissolved in 6 mL glacial acetic acid and 4 mL 6 M phosphoric acid) and 100 µL of glacial acetic acid for 1 h at 100 °C. The reaction was stopped by incubation on ice for 5 min, and the mixture was extracted with 200 µL toluene. The toluene phase was separated, and absorbance at 520 nm was used to determine the concentration of proline. All the reactions were performed in triplicates and a standard curve ranging in concentrations of 0.05–1 mM proline was generated for determining proline concentration of samples.

### Western blot analyses

Cells were treated with various concentrations of METH for 24 h, after which cells were harvested and washed with PBS. Cell lysates were prepared as per our published method^50^ and quantified according to standard BCA protein assay (Pierce, USA). Equal amounts of cell lysates (20 µg) were resolved on SDS–polyacrylamide gels and transferred to nitrocellulose membranes using a semi-dry blotter (Bio-Rad). Membranes were blocked using Tris-buffered saline with 5% nonfat milk (pH 8.0; Sigma). Blots were then probed with the appropriate primary antibody in blocking buffer overnight at 4 °C. Incubation with secondary anti-mouse or anti-rabbit IgG antibodies conjugated to horseradish peroxidase (1:2000) was performed at room temperature for 1 h. All blots were washed in Tris-buffered saline with Tween 20 (pH 8.0; Sigma) and developed using the enhanced chemiluminescence (ECL) procedure (BioRad). Blots were routinely stripped by Restore Plus Stripping Buffer (Pierce) and reprobed with anti-actin monoclonal antibody to serve as loading controls. The density of the band was evaluated by Bio-Rad imaging-lab 4.0 software (https://www.bio-rad.com/en-pl/product/image-lab-software?ID=KRE6P5E8Z).

### Transfection of *P5CS* expression construct in CHO-K1 cells

CHO-K1 cells (1 × 10^5^) were seeded per well in 6-well plates for 24 h in complete F12K media. Prior to transfection, the cells were pretreated with METH for 1 h and then transfected with pcDNA control vector or P5CS expression vector. Transfections were performed with JetPrime (Polyplus) according to the manufacturer’s instructions. 24 h post transfection the cells were harvested by scraping. The cell supernatants and pellets were collected by centrifugation. Supernatants were assayed for glutamate and the cell pellets were analyzed by western blot as described before.

### Animal studies

All protocols were approved by the University of Florida and Vanderbilt University Institutional Animal Care and Use Committee (IACUC) policies and adhered to the NIH guidelines. WT C57BL/6J male mice were obtained from Jackson Laboratories (Vanderbilt; Stock No. 000664) or from the University of Florida Animal Care Services and were maintained on a 12-h light/dark cycle with food and water available ad libitum in their home cages. The mice were injected with a single dose of METH at 2 mg/kg body weight (acute exposure). Control mice received saline injections. The animals were euthanized after 24 h and whole brain were extracted, rapidly rinsed with ice-cold PBS and immediately flash frozen. Punches from the cortical regions were taken for each sample and homogenized manually in a sucrose buffer system supplemented with protease inhibitors. Samples were spun down at high speed for 5 min, the supernatants were transferred into separate tubes and the protein concentration was estimated by BCA assay (Pierce).

For brain slices studies 6-week-old male C57BL/6J mice were euthanized under isoflurane anesthesia after which 300-µm coronal brain slices containing the frontal cortex were prepared from whole brain tissue using a Leica Vibratome in oxygenated (95% O2; 5% CO2) ice-cold artificial cerebrospinal fluid (aCSF, in mM: 119 NaCl, 2.5 KCl, 1.3 MgCl2–6H2O, 2.5 CaCl2–2H2O, 1.0 NaH2PO4–H2O, 26.2 NaHCO3, and 11 glucose). Following a 30-min recovery period, slices were then incubated in either 200 µM amphetamine, 200 µM methamphetamine or vehicle (phosphate buffered saline, PBS). Following a 6-h incubation, cortical enriched tissue was isolated and flash frozen until processing for immunoblot analysis.

### Statistical analyses

Data are expressed as mean ± SEM obtained from at least three independent experiments. Significance of differences between control and treated samples was determined by Student’s t test. Values of p < 0.05 were considered to be statistically significant.

## Supplementary Information

Supplementary Information.

## References

[1] Barr AM (2006). The need for speed: An update on methamphetamine addiction. J. Psychiatry Neurosci..

[2] Vearrier D, Greenberg MI, Miller SN, Okaneku JT, Haggerty DA (2012). Methamphetamine: History, pathophysiology, adverse health effects, current trends, and hazards associated with the clandestine manufacture of methamphetamine. Dis. Month..

[3] Brecht ML, Greenwell L, Anglin MD (2007). Substance use pathways to methamphetamine use among treated users. Addict. Behav..

[4] Harada T, Tsutomi H, Mori R, Wilson DB (2018). Cognitive-behavioural treatment for amphetamine-type stimulants (ATS)-use disorders. Cochrane Database Syst. Rev..

[5] Chooi WT (2017). Early initiation of amphetamine-type stimulants (ATS) use associated with lowered cognitive performance among individuals with co-occurring opioid and ATS use disorders in Malaysia. J. Psychoact. Drugs.

[6] Rothman RB, Baumann MH (2003). Monoamine transporters and psychostimulant drugs. Eur. J. Pharmacol..

[7] Kish SJ (2008). Pharmacologic mechanisms of crystal meth. CMAJ.

[8] Lin M, Sambo D, Khoshbouei H (2016). Methamphetamine regulation of firing activity of dopamine neurons. J. Neurosci..

[9] Bennett BA, Hollingsworth CK, Martin RS, Harp JJ (1998). Methamphetamine-induced alterations in dopamine transporter function. Brain Res..

[10] Moszczynska A, Callan SP (2017). Molecular, behavioral, and physiological consequences of methamphetamine neurotoxicity: Implications for treatment. J. Pharmacol. Exp. Ther..

[11] Volz TJ, Hanson GR, Fleckenstein AE (2007). The role of the plasmalemmal dopamine and vesicular monoamine transporters in methamphetamine-induced dopaminergic deficits. J. Neurochem..

[12] Underhill SM (2014). Amphetamine modulates excitatory neurotransmission through endocytosis of the glutamate transporter EAAT3 in dopamine neurons. Neuron.

[13] Stephans SE, Yamamoto BK (1994). Methamphetamine-induced neurotoxicity: Roles for glutamate and dopamine efflux. Synapse.

[14] Mark KA, Soghomonian JJ, Yamamoto BK (2004). High-dose methamphetamine acutely activates the striatonigral pathway to increase striatal glutamate and mediate long-term dopamine toxicity. J. Neurosci..

[15] Zhou Y, Danbolt NC (2014). Glutamate as a neurotransmitter in the healthy brain. J. Neural Transm. (Vienna).

[16] Niciu MJ, Kelmendi B, Sanacora G (2012). Overview of glutamatergic neurotransmission in the nervous system. Pharmacol. Biochem. Behav..

[17] Meldrum BS (2000). Glutamate as a neurotransmitter in the brain: Review of physiology and pathology. J. Nutr..

[18] McEntee WJ, Crook TH (1993). Glutamate: Its role in learning, memory, and the aging brain. Psychopharmacology.

[19] Peng L (1993). Utilization of glutamine and of TCA cycle constituents as precursors for transmitter glutamate and GABA. Dev. Neurosci..

[20] Yudkoff M (1993). Brain glutamate metabolism: Neuronal-astroglial relationships. Dev. Neurosci..

[21] Daikhin Y, Yudkoff M (2000). Compartmentation of brain glutamate metabolism in neurons and glia. J. Nutr..

[22] Hertz L (2013). The glutamate-glutamine (GABA) cycle: Importance of late postnatal development and potential reciprocal interactions between biosynthesis and degradation. Front. Endocrinol. (Lausanne).

[23] Palmada M, Centelles JJ (1998). Excitatory amino acid neurotransmission. Pathways for metabolism, storage and reuptake of glutamate in brain. Front. Biosci..

[24] Choi DW (1988). Glutamate neurotoxicity and diseases of the nervous system. Neuron.

[25] Phang JM, Liu W, Hancock C, Christian KJ (2012). The proline regulatory axis and cancer. Front. Oncol..

[26] Phang JM (1985). The regulatory functions of proline and pyrroline-5-carboxylic acid. Curr. Top. Cell Regul..

[27] Phang JM (2019). Proline metabolism in cell regulation and cancer biology: recent advances and hypotheses. Antioxid. Redox Signal..

[28] Phang JM, Donald SP, Pandhare J, Liu Y (2008). The metabolism of proline, a stress substrate, modulates carcinogenic pathways. Amino Acids.

[29] Phang JM, Pandhare J, Liu Y (2008). The metabolism of proline as microenvironmental stress substrate. J. Nutr..

[30] Wyse AT, Netto CA (2011). Behavioral and neurochemical effects of proline. Metab. Brain Dis..

[31] Fremeau RT, Caron MG, Blakely RD (1992). Molecular cloning and expression of a high affinity L-proline transporter expressed in putative glutamatergic pathways of rat brain. Neuron.

[32] Cohen SM, Nadler JV (1997). Proline-induced potentiation of glutamate transmission. Brain Res..

[33] Martin D, Ault B, Nadler JV (1992). NMDA receptor-mediated depolarizing action of proline on CA1 pyramidal cells. Eur. J. Pharmacol..

[34] Pace JR, Martin BM, Paul SM, Rogawski MA (1992). High concentrations of neutral amino acids activate NMDA receptor currents in rat hippocampal neurons. Neurosci. Lett..

[35] Paterlini M (2005). Transcriptional and behavioral interaction between 22q11.2 orthologs modulates schizophrenia-related phenotypes in mice. Nat. Neurosci..

[36] Kanwar YS, Krakower CA, Manaligod JR, Justice P, Wong PW (1975). Biochemical, morphological and hybrid studies in hyperprolinemic mice. Biomedicine.

[37] Hayward DC (1993). The sluggish-A gene of Drosophila melanogaster is expressed in the nervous system and encodes proline oxidase, a mitochondrial enzyme involved in glutamate biosynthesis. Proc. Natl. Acad. Sci. U.S.A..

[38] Roussos P, Giakoumaki SG, Bitsios P (2009). A risk PRODH haplotype affects sensorimotor gating, memory, schizotypy, and anxiety in healthy male subjects. Biol. Psychiatry.

[39] Jacquet H (2005). Hyperprolinemia is a risk factor for schizoaffective disorder. Mol. Psychiatry.

[40] Clelland CL (2011). Evidence for association of hyperprolinemia with schizophrenia and a measure of clinical outcome. Schizophr. Res..

[41] Oresic M (2011). Metabolome in schizophrenia and other psychotic disorders: A general population-based study. Genome Med..

[42] Xicoy H, Wieringa B, Martens GJ (2017). The SH-SY5Y cell line in Parkinson's disease research: A systematic review. Mol. Neurodegener..

[43] Filograna R (2015). Analysis of the catecholaminergic phenotype in human SH-SY5Y and BE(2)-M17 neuroblastoma cell lines upon differentiation. PLoS ONE.

[44] Kovalevich J, Langford D (2013). Considerations for the use of SH-SY5Y neuroblastoma cells in neurobiology. Methods Mol. Biol..

[45] Kang Y (2019). Epicatechin prevents methamphetamine-induced neuronal cell death via inhibition of ER stress. Biomol. Ther. (Seoul).

[46] Park JH (2017). Asiatic acid attenuates methamphetamine-induced neuroinflammation and neurotoxicity through blocking of NF-kB/STAT3/ERK and mitochondria-mediated apoptosis pathway. J. Neuroinflamm..

[47] Melega WP, Cho AK, Harvey D, Lacan G (2007). Methamphetamine blood concentrations in human abusers: Application to pharmacokinetic modeling. Synapse.

[48] Kalasinsky KS (2001). Regional distribution of methamphetamine in autopsied brain of chronic human methamphetamine users. Forens. Sci. Int..

[49] De Ingeniis J (2012). Functional specialization in proline biosynthesis of melanoma. PLoS ONE.

[50] Dash S (2017). Poly (ADP-Ribose) polymerase-1 (PARP-1) induction by cocaine is post-transcriptionally regulated by miR-125b. eNeuro..

[51] Pandhare J, Donald SP, Cooper SK, Phang JM (2009). Regulation and function of proline oxidase under nutrient stress. J. Cell Biochem..

[52] Bates LS, Waldren RP, Teare ID (1973). Rapid determination of free proline for water-stress studies. Plant Soil.

[53] McDonnell-Dowling K, Kelly JP (2017). The role of oxidative stress in methamphetamine-induced toxicity and sources of variation in the design of animal studies. Curr. Neuropharmacol..

[54] Yang X (2018). The main molecular mechanisms underlying methamphetamine-induced neurotoxicity and implications for pharmacological treatment. Front. Mol. Neurosci..

[55] Ramkissoon A, Wells PG (2015). Methamphetamine oxidative stress, neurotoxicity, and functional deficits are modulated by nuclear factor-E2-related factor 2. Free Radic. Biol. Med..

[56] Ozkan ED, Ueda T (1998). Glutamate transport and storage in synaptic vesicles. Jpn. J. Pharmacol..

[57] Herzog E (2004). Localization of VGLUT3, the vesicular glutamate transporter type 3, in the rat brain. Neuroscience.

[58] Barger CJ, Branick C, Chee L, Karpf AR (2019). Pan-cancer analyses reveal genomic features of FOXM1 overexpression in cancer. Cancers (Basel).

[59] Kao FT, Puck TT (1967). Genetics of somatic mammalian cells. IV. Properties of Chinese hamster cell mutants with respect to the requirement for proline. Genetics.

[60] Smith RJ, Downing SJ, Phang JM, Lodato RF, Aoki TT (1980). Pyrroline-5-carboxylate synthase activity in mammalian cells. Proc. Natl. Acad. Sci. U.S.A..

[61] McFarland K, Lapish CC, Kalivas PW (2003). Prefrontal glutamate release into the core of the nucleus accumbens mediates cocaine-induced reinstatement of drug-seeking behavior. J. Neurosci..

[62] Schwartz TL, Sachdeva S, Stahl SM (2012). Glutamate neurocircuitry: Theoretical underpinnings in schizophrenia. Front. Pharmacol..

[63] Stephans SE, Yamamoto BY (1995). Effect of repeated methamphetamine administrations on dopamine and glutamate efflux in rat prefrontal cortex. Brain Res..

[64] Maragakis NJ, Rothstein JD (2001). Glutamate transporters in neurologic disease. Arch. Neurol..

[65] Kalivas PW, Volkow ND (2011). New medications for drug addiction hiding in glutamatergic neuroplasticity. Mol. Psychiatry.

[66] Sugimoto K (2015). A clinically attainable dose of L-asparaginase targets glutamine addiction in lymphoid cell lines. Cancer Sci..

[67] Burnett EJ, Chandler LJ, Trantham-Davidson H (2016). Glutamatergic plasticity and alcohol dependence-induced alterations in reward, affect and cognition. Prog. Neuropsychopharmacol. Biol. Psychiatry.

[68] Borjkhani M, Bahrami F, Janahmadi M (2018). Computational modeling of opioid-induced synaptic plasticity in hippocampus. PLoS ONE.

[69] Earle ML, Davies JA (1991). The effect of methamphetamine on the release of glutamate from striatal slices. J. Neural Transm. Gen. Sect..

[70] Pereira FC (2012). Disruption of striatal glutamatergic/GABAergic homeostasis following acute methamphetamine in mice. Neurotoxicol. Teratol..

[71] Melega WP (2008). Long-term methamphetamine administration in the vervet monkey models aspects of a human exposure: Brain neurotoxicity and behavioral profiles. Neuropsychopharmacology.

[72] Bak LK, Schousboe A, Waagepetersen HS (2006). The glutamate/GABA-glutamine cycle: Aspects of transport, neurotransmitter homeostasis and ammonia transfer. J. Neurochem..

[73] Wojcik SM (2004). An essential role for vesicular glutamate transporter 1 (VGLUT1) in postnatal development and control of quantal size. Proc. Natl. Acad. Sci. U.S.A..

[74] Wilson NR (2005). Presynaptic regulation of quantal size by the vesicular glutamate transporter VGLUT1. J. Neurosci..

[75] Zhu L (2016). mRNA changes in nucleus accumbens related to methamphetamine addiction in mice. Sci. Rep..

[76] Kim M (2019). Current understanding of methamphetamine-associated metabolic changes revealed by the metabolomics approach. Metabolites.

[77] Narita M (2008). Post-synaptic action of morphine on glutamatergic neuronal transmission related to the descending antinociceptive pathway in the rat thalamus. J. Neurochem..

[78] Kim J, Ham S, Hong H, Moon C, Im HI (2016). Brain reward circuits in morphine addiction. Mol. Cells.

[79] Moron JA (2007). Morphine administration alters the profile of hippocampal postsynaptic density-associated proteins: A proteomics study focusing on endocytic proteins. Mol. Cell Proteomics.

[80] Phang JM, Liu W, Hancock CN, Fischer JW (2015). Proline metabolism and cancer: Emerging links to glutamine and collagen. Curr. Opin. Clin. Nutr. Metab. Care.

[81] Delwing D, Delwing D, Sanna RJ, Wofchuk S, Wyse AT (2007). Proline promotes decrease in glutamate uptake in slices of cerebral cortex and hippocampus of rats. Life Sci..

[82] Harris JJ, Jolivet R, Attwell D (2012). Synaptic energy use and supply. Neuron.

[83] Yellen G (2018). Fueling thought: Management of glycolysis and oxidative phosphorylation in neuronal metabolism. J. Cell Biol..

[84] Ashrafi G, Ryan TA (2017). Glucose metabolism in nerve terminals. Curr. Opin. Neurobiol..

[85] Liu W, Hancock CN, Fischer JW, Harman M, Phang JM (2015). Proline biosynthesis augments tumor cell growth and aerobic glycolysis: Involvement of pyridine nucleotides. Sci. Rep..

[86] Grinde MT (2019). Glutamine to proline conversion is associated with response to glutaminase inhibition in breast cancer. Breast Cancer Res..

[87] Kardos GR, Wastyk HC, Robertson GP (2015). Disruption of proline synthesis in melanoma inhibits protein production mediated by the GCN2 pathway. Mol. Cancer Res..

[88] Doench JG (2016). Optimized sgRNA design to maximize activity and minimize off-target effects of CRISPR-Cas9. Nat. Biotechnol..

