# Supplementary material for: Activation of proline biosynthesis is critical to maintain glutamate homeostasis during acute methamphetamine exposure
Source: Sci Rep. 2021 Jan 14;11:1422. doi: 10.1038/s41598-020-80917-7 (PMC7809342; doi:10.1038/s41598-020-80917-7)
Supplement: Supplementary file 1 — Supplementary Information. [file 41598_2020_80917_MOESM1_ESM.pdf]

Figure 1 (B) probed using Biorad Chemidoc

POX

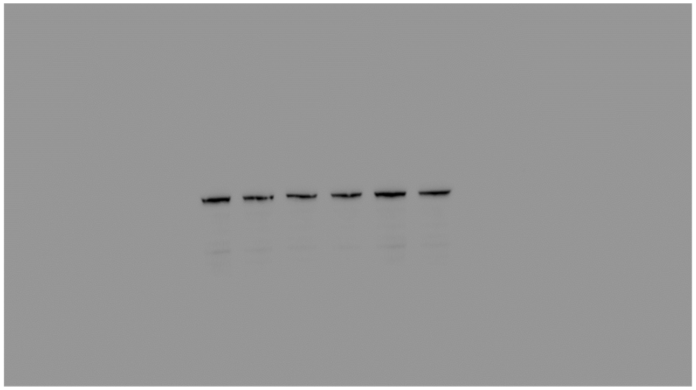

P5CDH

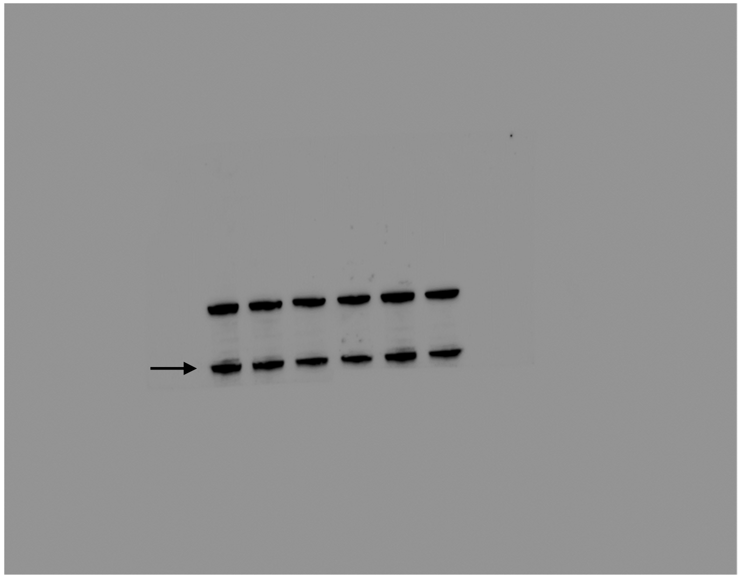

β-actin

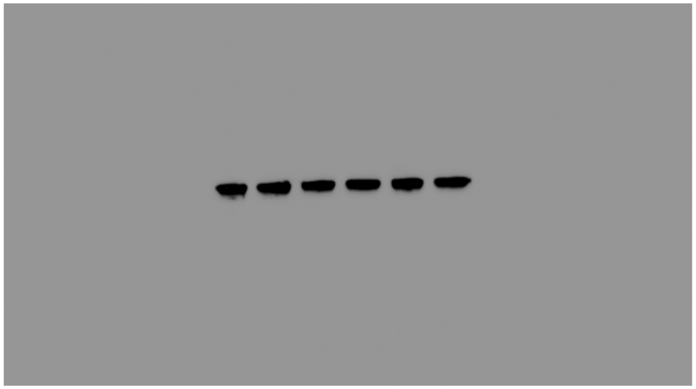

Figure 1 (E) probed using X-ray film

P5CS

β-actin

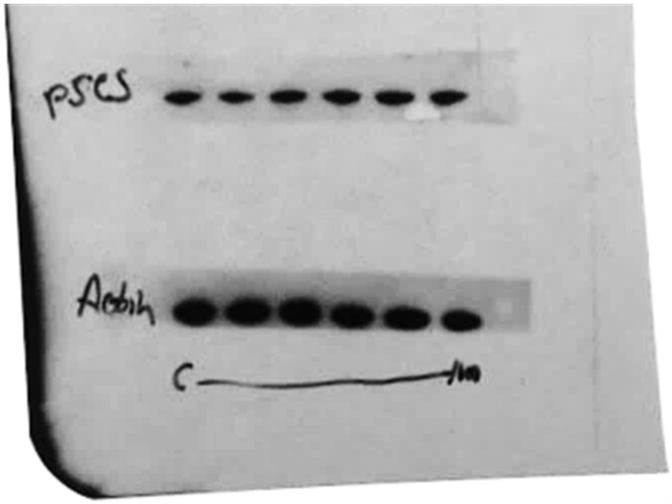

PYCR2

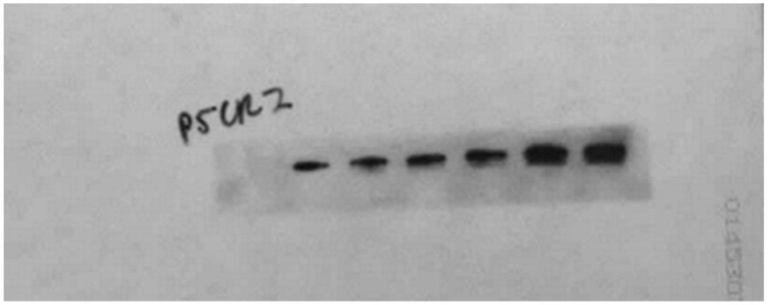

PYCR1

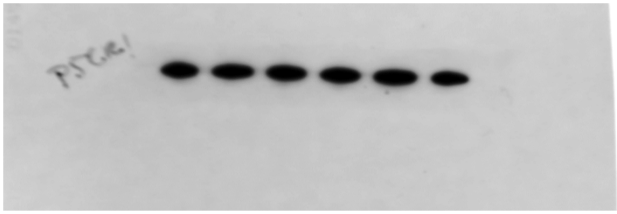

Figure 2 (A) probed using Biorad Chemidoc

Differentiated cells-METH

P5CS

PYCR2

β-actin

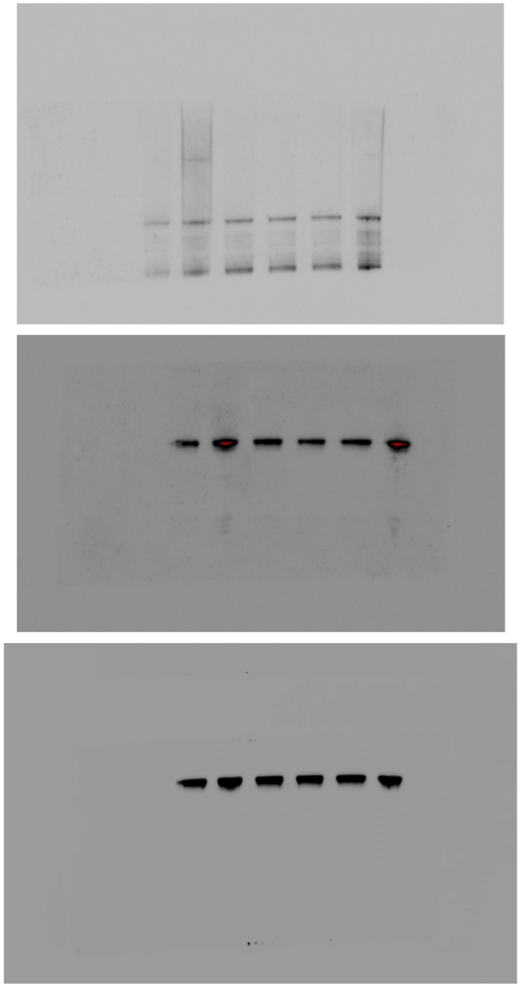

Figure 2 (D) probed using Biorad Chemidoc

Brain slices-METH

P5CS

PYCR2

β-actin

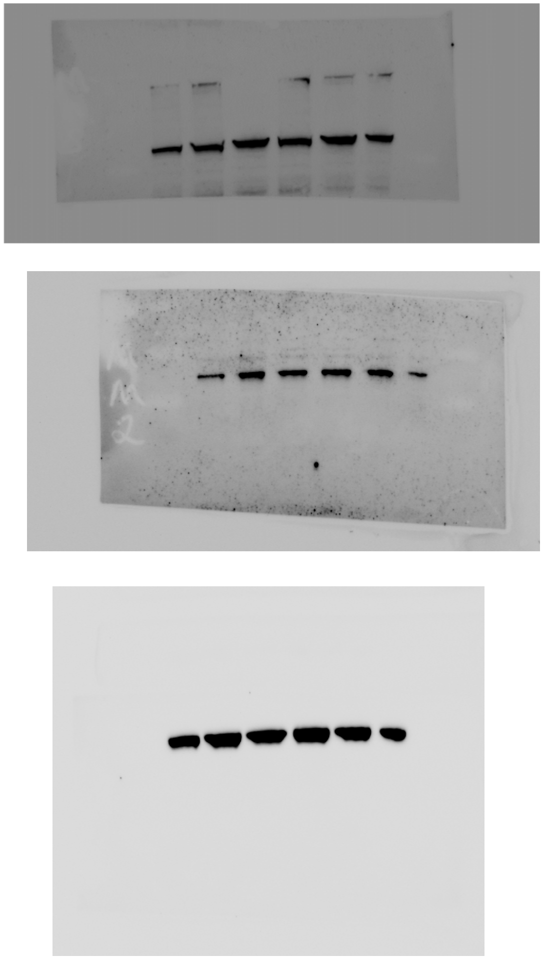

Figure 2 (G) probed using Biorad Chemidoc

undifferentiated cells-AMPH

P5CS

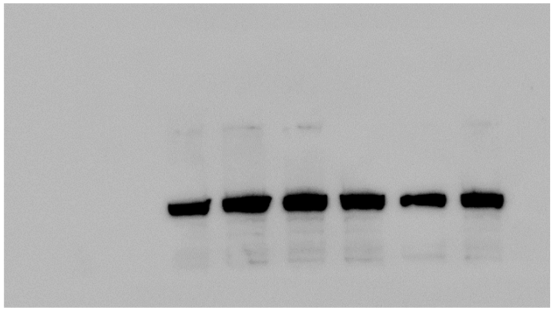

PYCR2

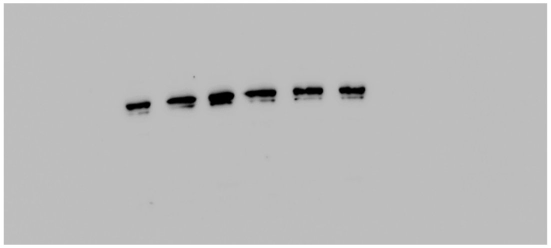

β-actin

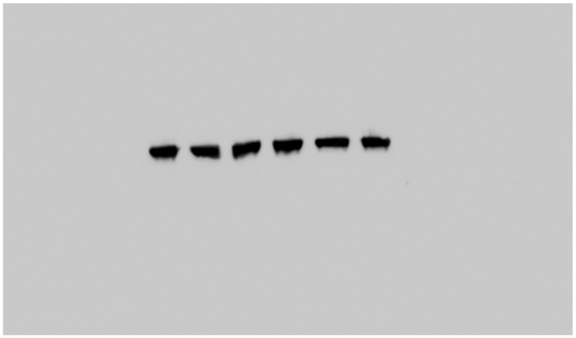

Figure 2 (J) probed using Biorad Chemidoc

Differentiated cells-AMPH

P5CS

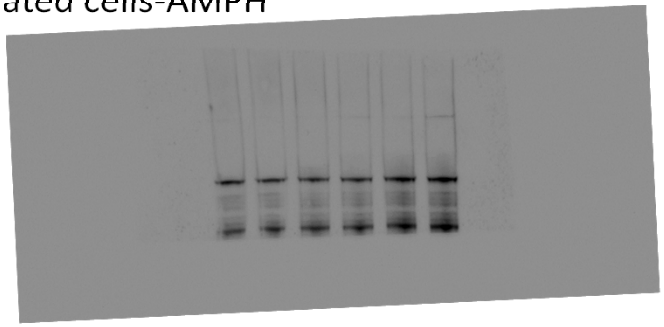

PYCR2

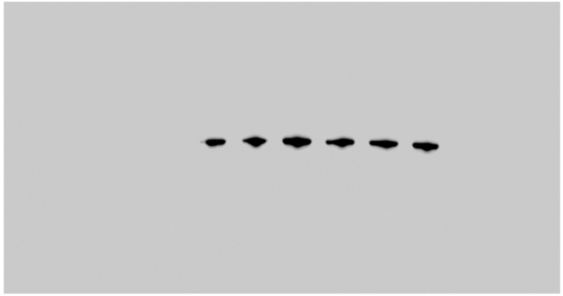

β-actin

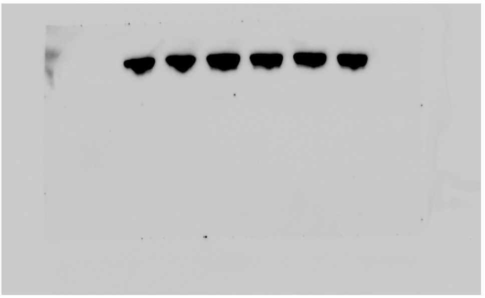

Figure 2 (M) probed using Biorad Chemidoc

Brain slices-AMPH

P5CS

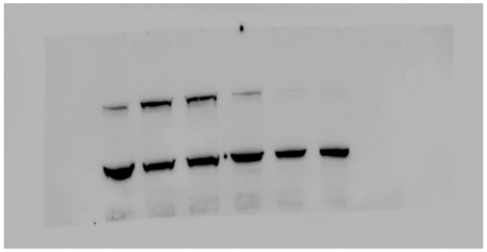

PYCR2

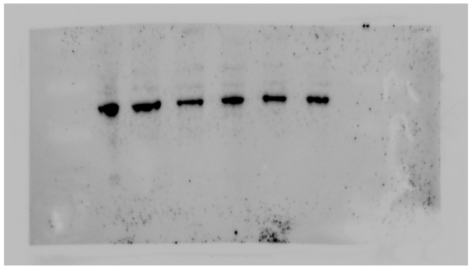

β-actin

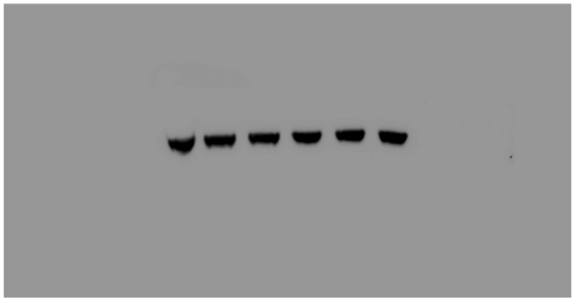

Figure 3 (D) probed using Biorad Chemidoc

GLS

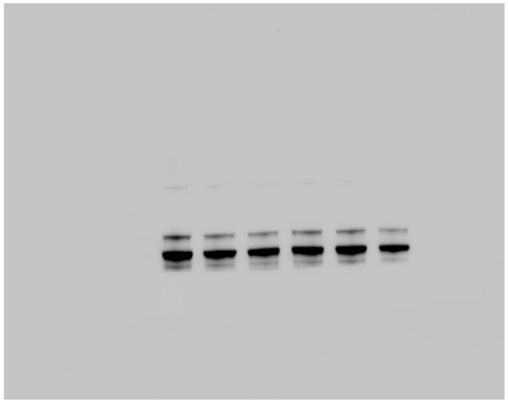

vGlut1

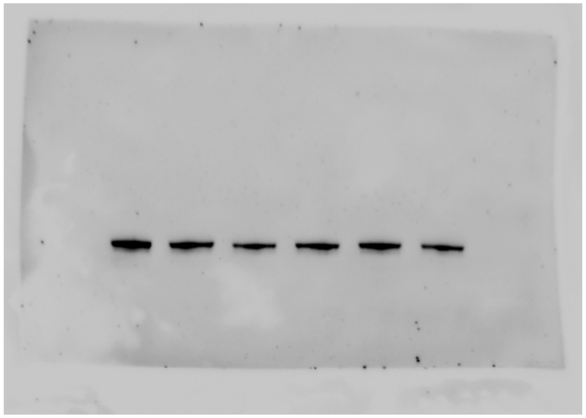

β-actin

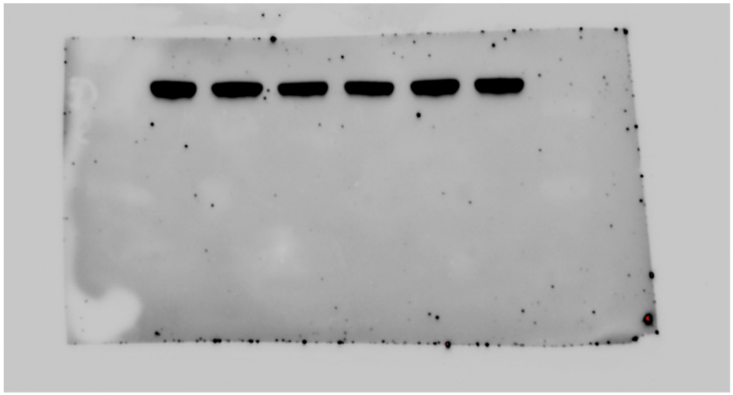

Figure 4 (A) probed using X-Ray film-WT vs KO

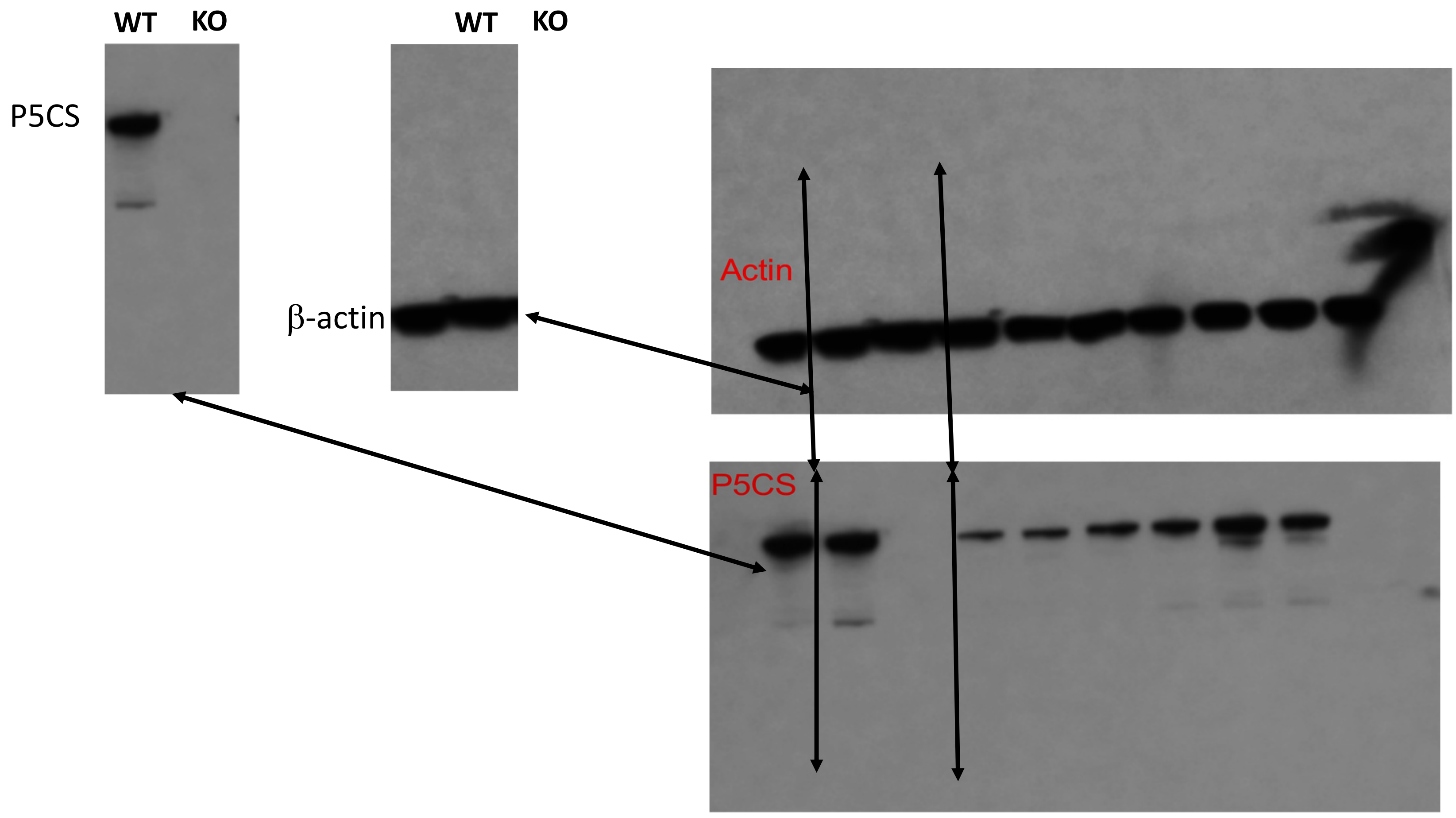

Blots were cut for two lanes only because the other samples are unrelated to this project

Figure 4 (D) probed using Biorad Chemidoc

PYCR2

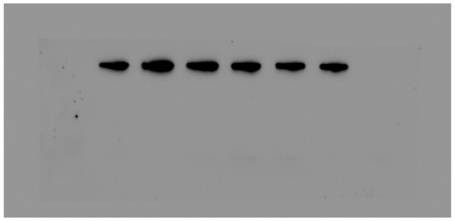

β-actin

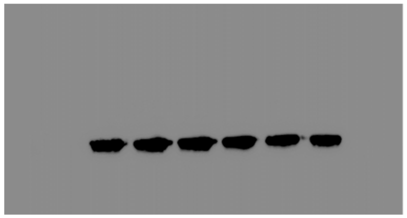

Figure 4 (F) probed using Biorad Chemidoc

GLS

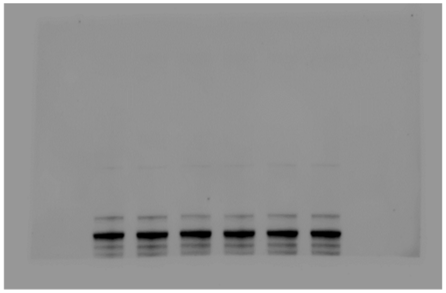

vGlut1

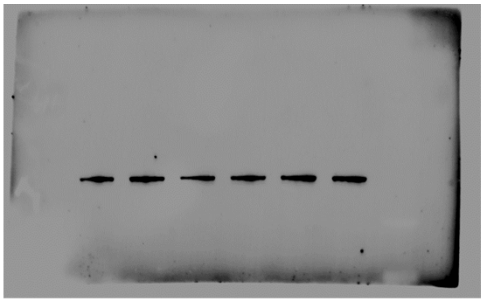

β-actin

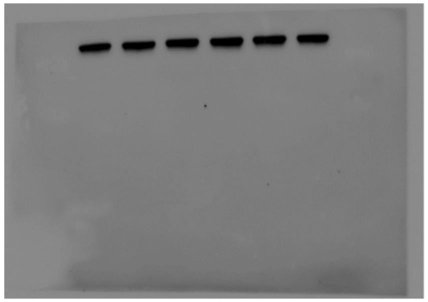

Figure 5 probed using X-ray film

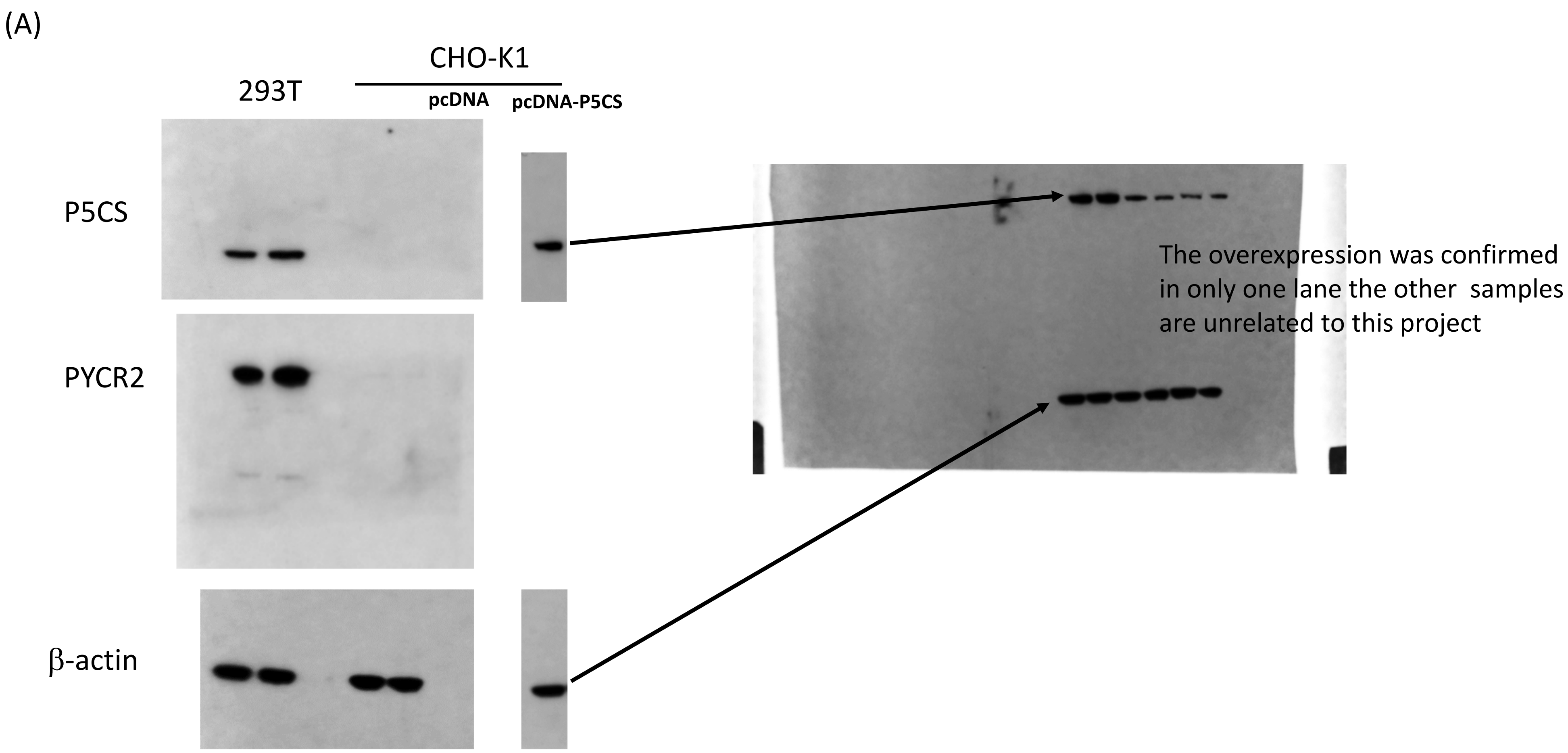

Mouse brain cortical regions

Figure 6 (A) probed using X-ray film

P5CS

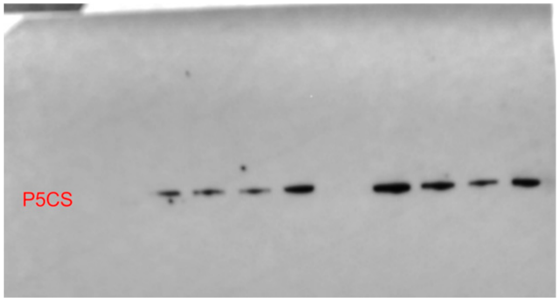

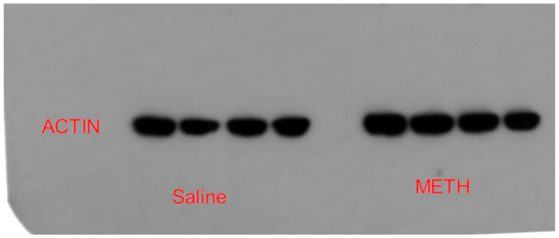

β-actin

Figure 6 (C) probed using X-ray film

PYCR2

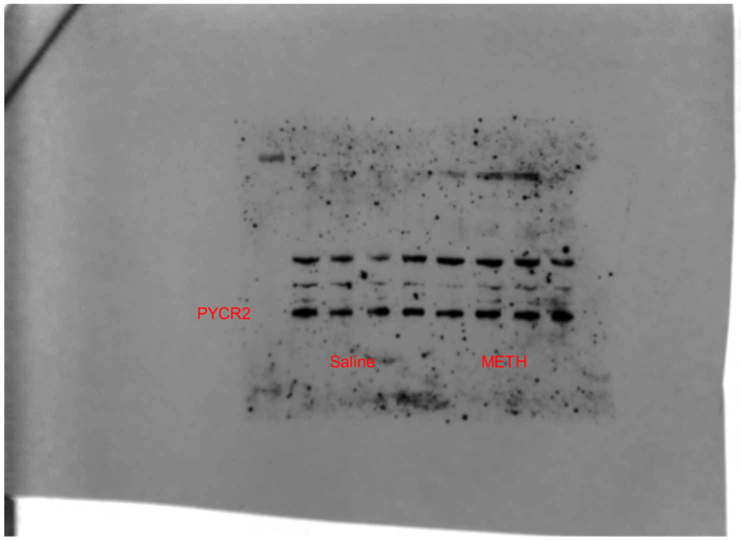

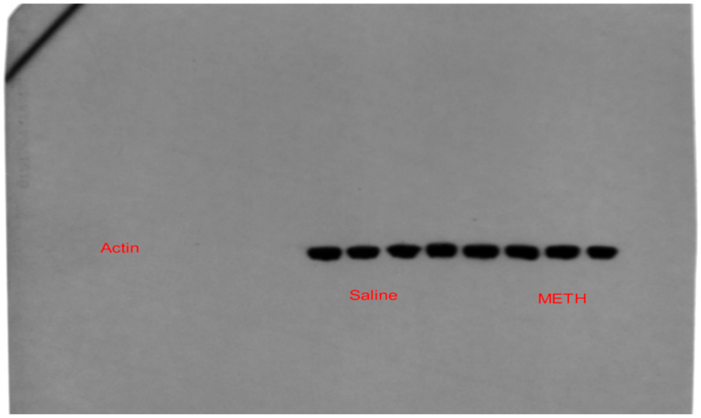

β-actin
